# NMDA receptor inhibition increases, synchronizes, and stabilizes the collective pancreatic beta cell activity: Insights through multilayer network analysis

**DOI:** 10.1371/journal.pcbi.1009002

**Published:** 2021-05-11

**Authors:** Marko Šterk, Lidija Križančić Bombek, Maša Skelin Klemen, Marjan Slak Rupnik, Marko Marhl, Andraž Stožer, Marko Gosak

**Affiliations:** 1 Faculty of Medicine, University of Maribor, Maribor, Slovenia; 2 Faculty of Natural Sciences and Mathematics, University of Maribor, Maribor, Slovenia; 3 Center for Physiology and Pharmacology, Medical University of Vienna, Vienna, Austria; 4 Alma Mater Europaea–ECM, Maribor, Slovenia; 5 Faculty of Education, University of Maribor, Maribor, Slovenia; University of Southern California, UNITED STATES

## Abstract

NMDA receptors promote repolarization in pancreatic beta cells and thereby reduce glucose-stimulated insulin secretion. Therefore, NMDA receptors are a potential therapeutic target for diabetes. While the mechanism of NMDA receptor inhibition in beta cells is rather well understood at the molecular level, its possible effects on the collective cellular activity have not been addressed to date, even though proper insulin secretion patterns result from well-synchronized beta cell behavior. The latter is enabled by strong intercellular connectivity, which governs propagating calcium waves across the islets and makes the heterogeneous beta cell population work in synchrony. Since a disrupted collective activity is an important and possibly early contributor to impaired insulin secretion and glucose intolerance, it is of utmost importance to understand possible effects of NMDA receptor inhibition on beta cell functional connectivity. To address this issue, we combined confocal functional multicellular calcium imaging in mouse tissue slices with network science approaches. Our results revealed that NMDA receptor inhibition increases, synchronizes, and stabilizes beta cell activity without affecting the velocity or size of calcium waves. To explore intercellular interactions more precisely, we made use of the multilayer network formalism by regarding each calcium wave as an individual network layer, with weighted directed connections portraying the intercellular propagation. NMDA receptor inhibition stabilized both the role of wave initiators and the course of waves. The findings obtained with the experimental antagonist of NMDA receptors, MK-801, were additionally validated with dextrorphan, the active metabolite of the approved drug dextromethorphan, as well as with experiments on NMDA receptor KO mice. In sum, our results provide additional and new evidence for a possible role of NMDA receptor inhibition in treatment of type 2 diabetes and introduce the multilayer network paradigm as a general strategy to examine effects of drugs on connectivity in multicellular systems.

## Author summary

Information processing in tissues is regulated by networks of interacting cells and through crosstalk with their environment. This is also true for islets of Langerhans in which several hundreds of beta cells are interconnected to ensure a proper secretion of insulin, a hormone crucial for the control of metabolic homeostasis. Since the loss of coordinated cellular activity is accompanied by a disruption of insulin secretion patterns, one of the main hallmarks of diabetes, it is of utmost importance to understand the underlying mechanisms that drive collective cellular activity in the islets and how these are affected in disease. Recently, NMDA receptor inhibitors were shown to boost beta cell activity and insulin secretion and were therefore suggested as potential antidiabetic drugs. However, their impact on the multicellular dynamics is not known. In the present study, we address this issue by combining high-frequency functional multicellular calcium imaging in pancreas tissue slices with network science approaches. Our findings reveal that NMDA receptor inhibition increases and synchronizes the activity of beta cell populations. Moreover, by considering individual intercellular calcium waves as network layers we show that NMDA receptor inhibition stabilizes the course of intercellular signals and confines the wave initiator regions.

## Introduction

Type 2 diabetes mellitus (T2DM) has become the most common metabolic disease in the developed world, representing a substantial public health threat with high personal and health-system costs [1]. Current treatment strategies are aiming at enhanced insulin secretion, insulin replacement, pharmacological or dietary improvement of insulin sensitivity, and direct plasma glucose reduction. Insulin is produced and secreted from beta cells in pancreatic islets. They are the dominant endocrine cell type, occupying 60–80% of the islet volume [2]. Insulin is the single most important anabolic and anti-catabolic hormone that coordinates postprandial storage and interprandial consumption of all energy-rich nutrients [3]. The main condition for insulin secretion from beta cells is sufficient plasma glucose concentration. At stimulatory plasma levels, glucose exerts pleiotropic effects on beta cells, ultimately leading to a raise in cytosolic Ca^2+^ concentration, which triggers and further regulates insulin release [4]. Additional neurohormonal pathways, involving protein-kinase A (PKA) and protein-kinase C (PKC)-dependent processes, can amplify the Ca^2+^-dependent pathway or independently activate insulin secretion [5,6].

With neighboring endocrine cells in an islet, beta cells form complex homo- and hetero-typic functional interactions [2,5,7–10]. There is strong evidence of beta cells interacting with each other as well as with other non-beta cells in their dynamical environment. An average murine islet of Langerhans contains several hundred beta cells that form a functional cell collective. The higher three-dimensional organization and interconnectedness of intrinsically nonlinear and highly heterogeneous beta cells is crucial for proper collective behavior and hormone secretion [11–15]. The electrical coupling through connexin36 (Cx36) gap junctions, possibly together with other modes of intercellular communication, has been described as the main synchronizing mechanism [16–22]. This ensures well-synchronized oscillatory membrane potential depolarizations and subsequent changes in [Ca^2+^]_ic_ that can spread across an islet [23,24], orchestrating the coordinated activity of cells and pulsatile insulin release [5,25,26]. Coupling is also recognized as an essential factor for confining beta cell heterogeneity and biological noise [12,19,27,28]. Conversely, alterations in islet morphology and disrupted intercellular communication pathways cause a loss of synchronized beta cell activity, leading to an impairment of normal oscillatory patterns of insulin secretion elicited by glucose, a defining characteristic of obesity and T2DM [9,12,19,29–33]. Importantly, different pharmacological substances for T2DM treatment may have different long-term effectiveness at least partly due to different effects they exert on intercellular coupling and survival [34,35].

In this regard, glutamate signaling via N-Methyl-D-Aspartate Receptors (NMDARs) has been shown to have an important role in beta cell stimulus-secretion coupling [35,36]. However, a complete mechanistic insight into its effects is still lacking. Currently, the main body of knowledge regarding the function of NMDARs stems from the studies on neurons, but also the number of studies presenting similar patterns of glutamate signaling in pancreatic beta cells is steadily increasing [37]. In the nervous system, NMDARs are involved in neuronal survival, brain plasticity and communication between cells. A dysfunction of NMDARs, such as altered subunit expression, trafficking, activity, or localization, can lead to detrimental cognitive impairments, such as Alzheimer’s disease, Parkinson’s disease, depression, schizophrenia, and autism [38]. In hippocampal slices, they mediate fast excitatory neurotransmission between neurons as well as neurotransmission events mediated by glutamate released from astrocytes and acting on extrasynaptic NMDA receptors [39]. Studies in neurons have also shown that NMDARs are able to activate K_ATP_ channels in subthalamic [16] and in dopamine midbrain neurons [40]. Furthermore, they are able to activate SK channels in dendritic spines [41].

K_ATP_ channels and SK channels have been reported to be present and affected by targeting of NMDARs also in pancreatic beta cells [35,42–45]. It has been shown that excessively high extracellular glutamate concentration may be detrimental for human islet β-cells [46], contributing to the development of diabetes through excessive activation of NMDARs in beta-cells, and accelerating their dysfunction and apoptosis induced by hyperglycemia [47]. This is consistent with the reports showing neuronal cell death after high-glutamate-mediated excitotoxicity via excessive activation of NMDARs in the CNS [48].

At more physiological concentrations, glutamate released from alpha and beta cells, or present in blood plasma and reaching pancreatic beta cells through the fenestrated endothelium at high enough concentrations to saturate NMDARs has been proposed to have an inhibitory effect on insulin secretion [36]. In experiments with human and mouse islets, inhibition of NMDARs resulted in an increase in glucose-stimulated insulin secretion and prolonged depolarization of beta cells during elevated [Ca^2+^]_ic_ in the plateau phase [35]. Given the described similarities between neurons and beta cells, one of the possible mechanisms to explain the observed effects of glutamate and NMDARs on insulin release would be the regulation of intercellular communication. It was namely shown that weak electrical coupling of neurons is strengthened by activation of NMDARs at and near synaptic loci [49], and that in cultured astrocytes glutamate induces [Ca^2+^]_c_ waves which propagate between adjacent astrocytes in confluent cultures establishing networks and mediating a long-range signaling system [50]. Furthermore, it has been shown that in the postnatal development of mammalian central nervous system, NMDARs are responsible for the developmental uncoupling of neuronal gap junctions, with the progressive downregulation of connexin 36 (Cx36) with increasing age. The gap junction uncoupling has been prevented with blockade of NMDARs [51].

Since uncoupling and downregulation of Cx36 also heavily affect communication between beta cells and their function, in the present paper we investigated how NMDAR inhibition affects the collective activity of beta cell collectives within tissue slices. To this end, we combined advanced high-frequency functional multicellular imaging using a novel stable and bright low-affinity Ca^2+^ probe with classical physiological analyses, as well as functional and multilayer network tools [13,25,52–57]. The latter have proven to be a much richer computational framework than classical network analyses and particularly suitable for the description of time-varying networks and for exploring the temporal robustness of functional connectivity patterns [25,58–63]. Representing the spreading Ca^2+^ waves in islets of Langerhans as network layers, to the best of our knowledge for the first time, enabled us to interpret the effects of NMDAR inhibition on the multicellular level of beta cell activity, far beyond classical physiological and network methods for cellular signaling analysis.

## Results

**Fig 1:**
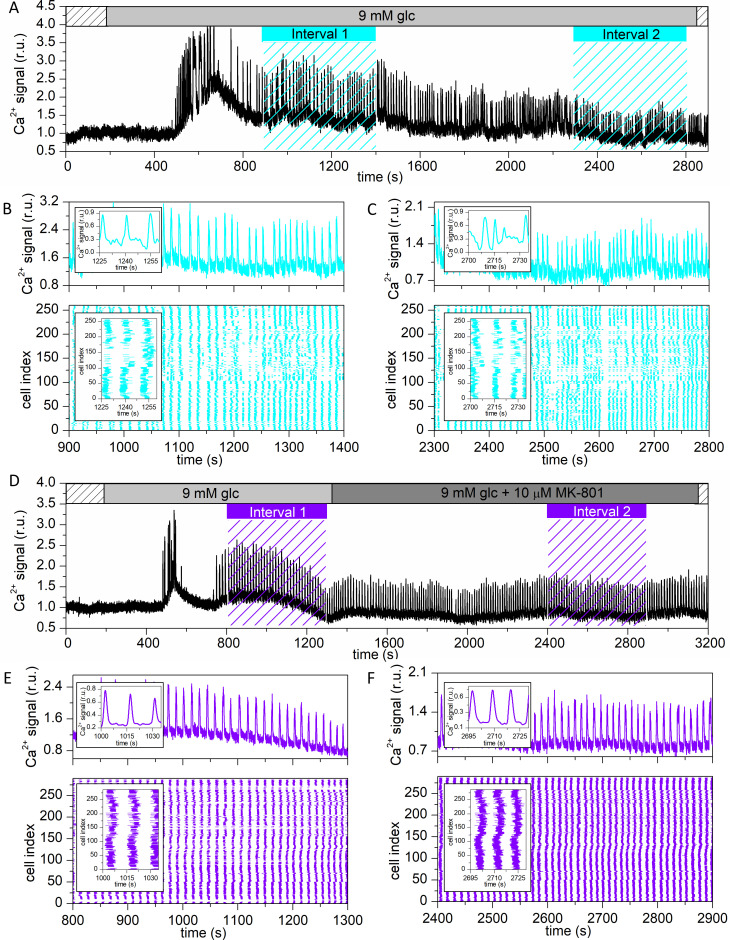
Visualizing beta cell activity in different stimulation protocols. Representative [Ca^2+^]_ic_ signals and raster plots in a recording with protocol G (9 mM glucose only) (A) and protocol MK (9 mM glucose + 10 μM MK-801) (D). The two periods of the MK protocol are indicated with light grey and dark grey bars, respectively. The sub-stimulatory period in 6 mM glucose is indicated with dashed bars. Intervals 1 and 2, which were used for further analysis, are indicated in cyan for protocol G and in violet for protocol MK. Panels (B)-(C) and (E)-(F) show outtakes of the calcium signal of a single cell (upper part) and the binarized signal of all cells in the islet (lower part) for both protocols, respectively.

Fig 1A and 1D shows typical [Ca^2+^]_ic_ traces for two representative cells subjected to protocols G (9 mM glucose only) and MK (9 mM glucose → 9 mM glucose + 10 μM MK-801), respectively. In both cases, we observed a time delay of several minutes before response onset after switching from sub-stimulatory 6 mM to stimulatory 9 mM glucose. After an initial transient burst of high activity, the cells entered a plateau phase of sustained activity. The time intervals 1 and 2 (cyan and violet bars with dashed lines) were selected for further analysis to capture the beta cell behavior in the beginning of the plateau phase (interval 1; glucose only) and after prolonged stimulation (interval 2; glucose or glucose+MK-801). To represent the collective activity in intervals of interest, we plotted raster plots of binarized calcium activity of all cells in the given islet, along with the representative calcium signal (Fig 1B, 1C, 1E and 1F). It appears that the [Ca^2+^]_ic_ signaling patterns are changed from interval 1 to interval 2 and that the nature of this change was different in protocol G compared to MK. To unveil the characteristics of these changes in more detail and in a quantitative manner, we systematically analyzed different aspects of spatiotemporal beta cell activity, separately for each protocol.

**Fig 2:**
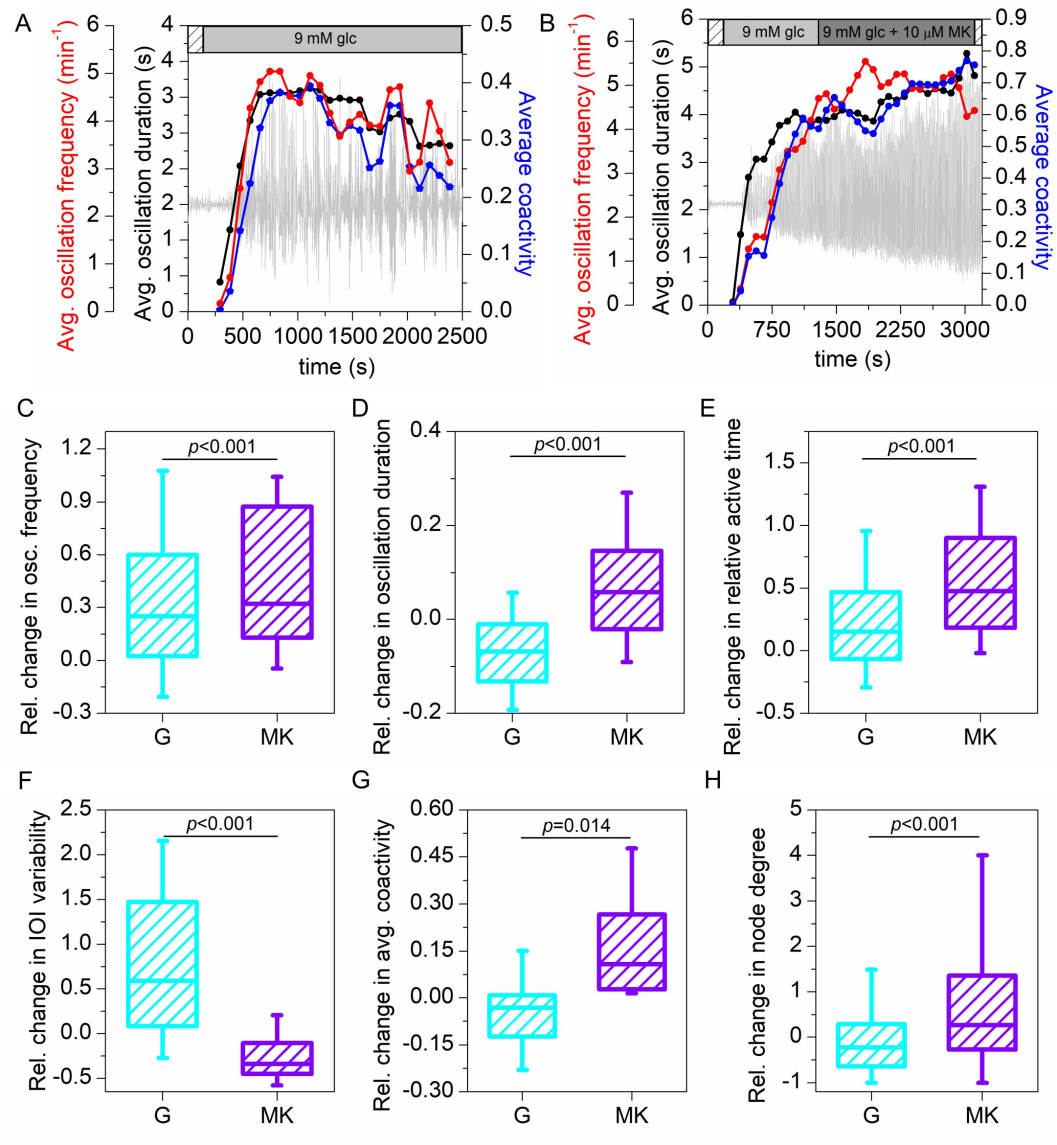
Quantifying beta cell activity in different protocols. Temporal evolution of the average oscillation frequency (red), average oscillation duration (black), and average coactivity of the cells (blue) for a recording in protocol G (A) and for a recording in protocol MK (B). Light grey curves show the average calcium signal. Panels (C)-(H) show the relative change in oscillation frequency, oscillation duration, relative active time, inter-oscillation interval variability, average coactivity, and node degree in the functional beta cell networks from interval 1 to interval 2 for all recordings in protocols G (cyan) and MK (violet). Whiskers indicate the 10th and 90th percentile, the box indicates the 25th and 75th percentile, and the horizontal line indicates the median value. Data were pooled from the following number of mice/cells/islets: 3/1373/10 (protocol G), 5/1731/12 (protocol MK). *p*–significance level. Statistical tests: Mann-Whitney Rank Sum Test (C, D, E, F, H) and Student’s t-test (G).

In Fig 2A and 2B we first presented the temporal evolution of the average oscillation frequency (red), average oscillation duration (black) and average coactivity (blue) for both protocols. In protocol G, we typically observed an increase of all signaling parameters when the cells responded to stimulation with glucose, followed by a rather continuous and moderate decrease of cellular activity and synchronicity (Fig 2A). In contrast, in protocol MK, the application of NMDAR inhibitor led to an increase of parameters–beta cells became on average more active and more synchronized (Fig 2B). To gain further insight, we presented in Fig 2C–2H the pooled data from multiple islets (10 islets for protocol G and 12 islets for protocol MK), showing the relative changes in individual parameters when comparing intervals 1 and 2. Evidently, the frequency of oscillations increased under prolonged stimulation in both protocols. The relative change in frequency was in both cases on average around 25–30% (Fig 2C). In contrast, the inhibition of NMDARs was characterized by significantly longer durations of oscillations compared to protocol G (Fig 2D). More specifically, while persistent glucose stimulation led in most of the islets to a decrease in duration, in protocol MK we detected an obvious increase in durations in the majority of the recordings, in accordance with our previous report [35]. Despite the decrease in oscillation duration in protocol G, there was an increase of relative active time from interval 1 to interval 2 in both protocols (Fig 2E). However, this increase was significantly and more than 2-fold higher in protocol MK. More specifically, the active time increased on average by approx. 20% in protocol G and by 50% in protocol MK. Interestingly, under the action of MK-801 the oscillations were more regular than in protocol G, where in the majority of the islets the oscillations became much more erratic in the second interval, as indicated with a positive change in inter-oscillation interval variability (Fig 2F). Finally, we assessed the beta cell synchronicity and functional connectivity by calculating the average coactivity and the node degrees in the functional beta cell networks. For protocol G, the change in average coactivity coefficient and node degree size was negative in most islets, whereas in protocol MK, beta cells became better synchronized and functionally connected in interval 2, reflected by a positive change of average coactivity and node degrees (Fig 2G and 2H). Apparently, NMDAR inhibition not only led to altered intracellular signaling in beta cells, reflected by changes in classical physiological parameters, but also considerably affected the collective activity of beta cell populations. Absolute values of the average signaling parameters (oscillation frequency, duration, inter-oscillation interval variability, and average coactivity) and their changes in each islet are given in S1 Fig.

**Fig 3:**
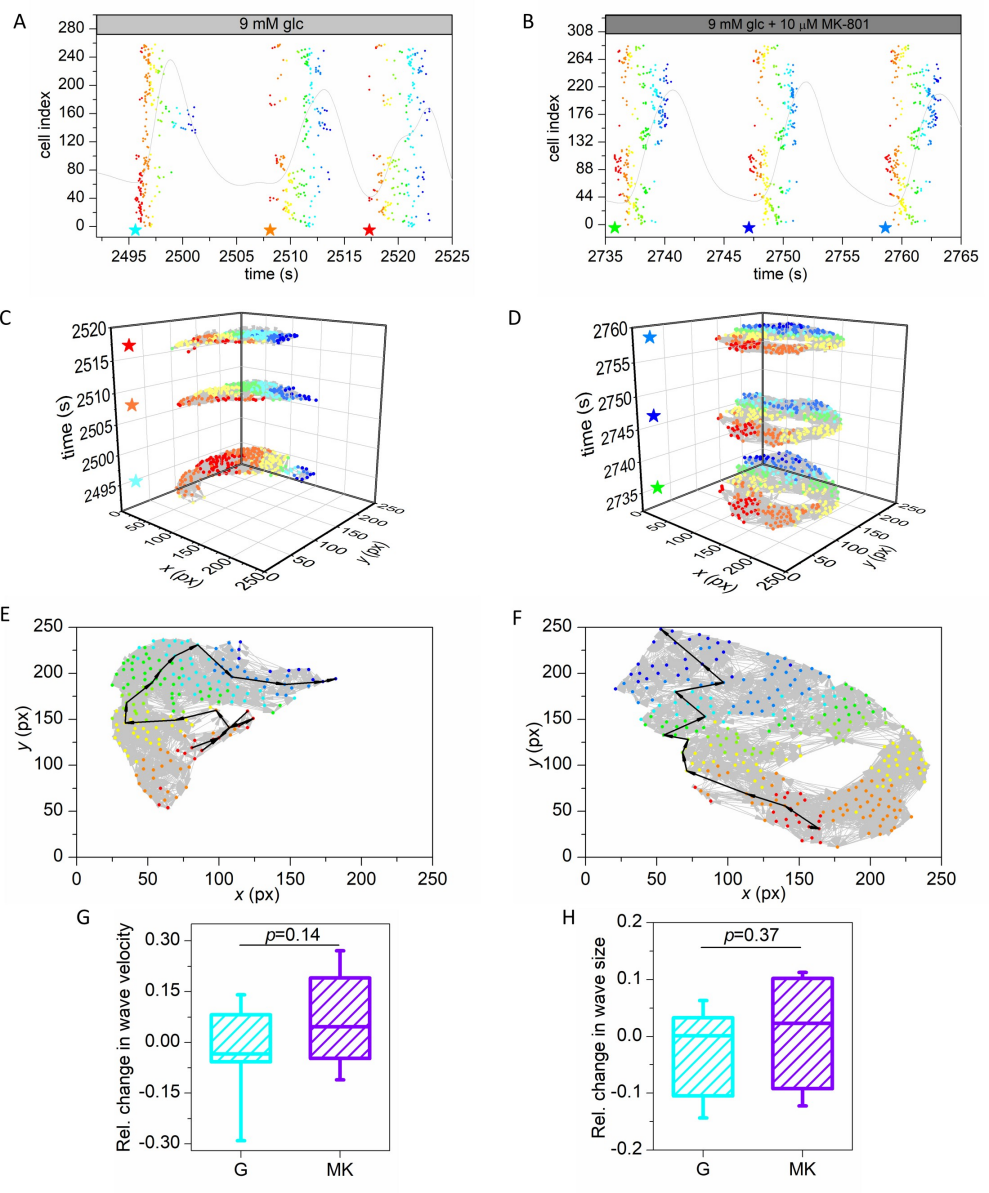
Multilayer network representation of calcium wave propagation in the pancreatic islets. Raster plot of activation times of cells for three detected calcium waves for a recording in protocol G (A), and a recording in protocol MK (B). Stars indicate the start times of waves and dots indicate the activation times of individual cells which participated in the wave. Colors of dots represent the activation times of cells within the individual wave, with red indicating initiator(s) and blue the cell(s) that activated last. Light grey curve represents the mean field calcium signal. Panels (C) and (D) are multilayer network representations of detected waves for the G and MK protocol, respectively. Stars indicate the start time of individual waves. Color coding is the same as in panels (A) and (B). Panels (E) and (F) show individual waves from a recording in the G and MK protocol, respectively. Light grey arrows are directed weighted connections between cells and the black bold arrows show the shortest paths from the initiator(s) to the cell(s) that activated last in the wave. Weights are the time delays between cells. Color coding is the same as in panels (A) and (B). Panels (G) and (F) show the relative change in wave velocity and relative wave size, from interval 1 to interval 2 for protocol G (cyan) and protocol MK (violet). Box-plots are defined the same as in Fig 2. Data were pooled from the following number of mice/cells/islets: 3/1280/9 (protocol G), 5/1622/11 (protocol MK). *p*–significance level. Statistical test: Student’s t-test (G, H).

Motivated by the notable effect of NMDAR inhibition on beta cell synchronicity, we next explored the calcium wave organization and communication patterns in islets in more detail. Fig 3A and 3B shows raster plots of three detected waves in interval 2 for both protocols. A star indicates the start of each detected wave and dots indicate activation times of individual cells, i.e., onsets of oscillations, within the same waves and are colored with respect to their order of activation, whereby red and blue color correspond to the first and last responding cell, respectively. To get a better insight into the spatiotemporal calcium dynamics, we displayed in Fig 3C and 3D the same calcium waves as in Fig 3A and 3B by means of multilayered networks, where each layer displays a particular wave. The cells are network nodes and are colored with the same color scale as in the raster plots above. The connections between cells are directed and the direction is determined by the difference in activation times, such that the connection is directed to the cell that is activated later and the weight of the connection represents the time lag. If a pair of cells was detected to activate simultaneously, an undirected connection was established (see Materials and Methods for details). This kind of visualization does not only allow us to track the spreading of the signal across the islet, but also enables us to scrutinize the characteristics of the intercellular communication patterns.

First, the network approach can be used to estimate the velocity of calcium wave propagation. In Fig 3E and 3F, one wave is shown for each protocol. Evidently, the waves did not follow a straight path from the first to the last responding cell. Therefore, to uncover the actual course, we calculated the weighted shortest path (black arrows) between the first and the last responder(s), which depicts the path of subsequent cellular activations, i.e., the course of the wave. The extracted geodesic path is then used to infer the traveled physical distance. Based on this, we calculated the velocity of the wave by considering the time lag between cells in the path and their physical distance. Importantly, to account for the effect of overestimating velocity in case the wave originated far below the focal plane, the first two connections from the first responder were discarded [24]. In other words, the velocity was calculated based on the shortest path from the third to the last responding cell in any given wave. In S2A Fig, the extracted values of calcium wave velocities are shown for all islets subjected to protocols G or MK, separately for intervals 1 and 2. In all cases, the velocity was on average around 90 μm/s, which is well in agreement with previous reports [4,23,24,64]. In Fig 3G, the relative changes of calcium wave velocity in individual islets are shown. It turned out that the signal propagation velocity remained approximately constant in both protocols. During NMDAR inhibition there was on average an increase and during incubation with glucose a slight decrease in wave velocity, but in the face of large inter-islet variability this change was not significant. Second, we quantified the changes in average calcium wave sizes. The results in Fig 3H reveal that in both protocols the average fractions of cells that were activated by the waves were approximately the same for both intervals. In S2B Fig, we additionally show the average relative wave sizes in individual islets in both intervals, separately for both protocols. Apparently, neither long-term exposure to stimulatory 9 mM glucose nor NMDAR inhibition considerably affected the average wave sizes. Altogether, the analyses presented in Fig 3 indicate that the decreased and increased beta cell synchronicity that were observed in protocols G and MK, respectively (Fig 2F), are most probably not due to changes in velocity or size of calcium waves. We therefore investigated the spatio-temporal organization of intercellular waves in further detail.

**Fig 4:**
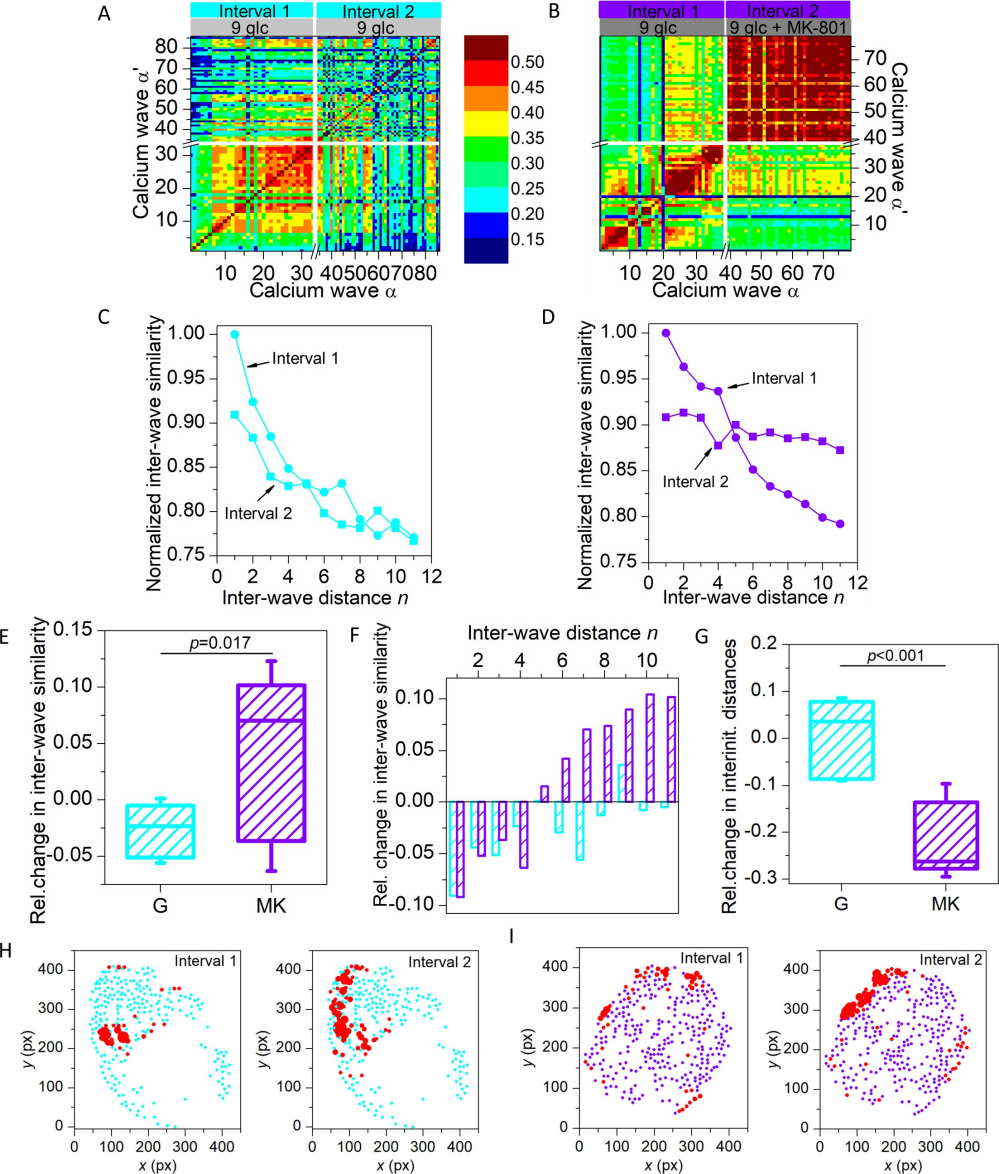
Assessing the inter-wave similarity and spatio-temporal stability of calcium waves. Inter-wave similarity matrices for all detected calcium waves in a recording in protocol G (A) and protocol MK (B) for interval 1 (light grey bar) and interval 2 (grey bar). Panels (C) and (D) show the normalized average inter-wave similarity of all recordings as a function of inter-wave distance for protocols G (cyan) and MK (violet), respectively. In both panels, interval 1 is shown with round symbols and interval 2 with square symbols, as indicated. In panel (E) the relative change in inter-wave similarity from interval 1 to interval 2 is presented for all recordings with protocol G (cyan) and protocol MK (violet). Panel (F) shows the relative change in inter-wave similarity from interval 1 to 2 as a function of distance for all the recordings in protocol G (cyan) and protocol MK (violet). In panel (G), the relative change in inter-initiator distances for all recordings in protocols G (cyan) and MK (violet) are shown. Panel (H) features representative islets for protocols G (cyan) and MK (violet). Red dots indicate wave initiators and the size of the dots corresponds to the fraction of times the cell initiated a wave. Box-plots are defined the same as in Fig 2. Data were pooled from the following number of mice/cells/islets: 3/1280/9 (protocol G), 5/1622/11 (protocol MK). *p*–significance level. Statistical test: Student’s t-test (E, G).

Third, it can be observed that for the three selected waves the course of the propagating signals was very variable for protocol G (Fig 3C), since the waves had different paths and started from different initiator regions (red colored regions). In contrast, for protocol MK the courses appeared to be more stable from wave to wave (Fig 3D). To extract and to quantify the general patterns of inter-wave similarity under different stimulation protocols, we calculated the similarity between network layers (see Materials and Methods). Fig 4A and 4B shows the inter-wave correlation matrices for representative recordings for protocols G and MK. For protocol G, altogether 86 calcium waves were detected (33 in interval 1 and 53 in interval 2). For protocol MK, 79 global waves were identified (38 in interval 1 and 41 in interval 2). Upon visual inspection of the matrix extracted for the recording in protocol G, it is clear that similarity between waves decreased in interval 2 (darker shades) compared to interval 1 (lighter shades). In contrast, in protocol MK the inter-wave similarity in interval 2 obviously increased, especially between waves that are more distant.

To investigate this observation into more detail, we show in Fig 4C and 4D the average inter-wave similarity as a function of inter-wave distance *n* for all recordings, separately for both intervals, normalized to the largest value in interval 1. Evidently, in interval 1 of both protocols the inter-wave similarity decreased with increasing inter-wave distance, which indicates that there was a tendency that waves directly following a given wave in general took more similar paths than waves occurring later on. Noteworthy, this tendency was preserved in interval 2 of protocol G, while in interval 2 of protocol MK, the inter-wave similarity decreased much less rapidly. The nearly constant inter-wave similarity during NMDAR inhibition indicates that the calcium waves exceedingly often followed almost exactly the same course. In Fig 4E, we show the relative changes in inter-wave similarity between intervals 2 and 1. For G, the average relative change in similarity between network layers was typically negative, whereas in MK, for the majority of islets the similarity between individual waves was significantly higher in interval 2. The increase was due to higher similarities between remote waves (Fig 4F). To visualize wave initiator regions, in Fig 4H we indicated wave initiators, i.e., pacemakers for both protocols and intervals in two typical islets. It appears that the wave initiators were more scattered in the second interval of protocol G compared to protocol MK. To quantify the heterogeneity of initiator regions, we computed the average distance between identified initiators. In Fig 4G, the relative change between the two intervals is shown for both protocols. It turned out that in protocol G the inter-initiator distances mostly increased, whereas in protocol MK the physical distance between initiator cells was always much lower in the second interval. The apparent stabilization of intercellular signal propagation by NMDAR inhibition is probably also the main reason for more regular oscillations in the second interval of protocol MK (Fig 2E), as well as for the observed increase in synchronicity (Fig 2G).

It should be noted that MK-801 that we employed in the present study is a selective NMDAR inhibitor that is used in experimental but not in clinical settings because of its severe adverse effects [35]. Therefore, we verified if comparable results are obtained with dextrorphan (DXO), the main *in vivo* metabolite of the NMDAR antagonist dextromethorphan, which has been already used in two placebo-controlled clinical trials [35,65]. S4A–S4C Fig presents the behavior of a typical islet stimulated with 9 mM glucose + 10 μm DXO (protocol DXO), whereas in S5 Fig we show the pooled data from 6 islets subjected to this protocol and compare the data with the results from other protocols. DXO had a very similar effect as MK-801, as it increased the frequency, the duration of the oscillations, the coherence of the oscillations, as well as beta cell synchronicity.

Additionally, to further elucidate the mode of action of NMDAR inhibition and since NMDAR inhibitors could be used in conjunction with cAMP elevating agents [65], we compared the effects of MK-801 and DXO with the effects of the cAMP elevating diterpene forskolin, which directly activates adenylate cyclase, at a concentration of 10 μM, both in independent experiments (S3 Fig) and as an add-on to DXO (S6 Fig). The behavior in a typical islet stimulated with 10 μm forskolin (protocol FOR) is visualized in S4D–S4F Fig, whereas in S5 Fig, we compared the data pooled from 5 islets subjected to protocol FOR with other three protocols (G, MK, and DXO). It can be observed that forskolin, in contrast to NMDAR inhibitors, decreased the durations of oscillations significantly, making them even shorter than in the second interval with glucose stimulation only, but at the same time, it profoundly increased the oscillation frequency. As a result, the median relative increase in active time was immense, i.e., by 140%, whilst in the other protocols the gains were around 20% (G), 50% (MK), and 70% (DXO). This finding is consistent with the view that NMDAR inhibition reduces the Ca^2+^ current that induces the SK4 (and possibly K_ATP_) channel-mediated repolarizing K^+^ current, thus increasing both burst duration and the frequency of electrical bursts [16,35,36,66–69] and Ca^2+^ oscillations. In contrast, cAMP may act to decrease K_ATP_ conductance, increase depolarizing leak current (via modulation of TRPM channels), and conductance of L-type Ca^2+^ channels, thus increasing the rate of inter-burst depolarization and the frequency of bursts and Ca^2+^ oscillations [70]. At the same time, it may act potently to decrease the duration of Ca^2+^ oscillations by increasing the repolarizing Ca^2+^-dependent K^+^ current via the larger influx of Ca^2+^ via the plasma membrane and possibly from intracellular stores [71–74]. Moreover, forskolin was found to positively affect collective beta cell activity; the increase in synchronicity was even higher when compared to the effect of NMDAR inhibitors. Given that we found no evidence for NMDAR inhibition influencing gap junctional coupling (no effect on wave velocity and wave size), the explanation for the greater synchronicity under these conditions lies in the greater stability of initiators, wave paths and inter-oscillation intervals. In contrast, cAMP is most probably able to increase gap junctional coupling via modulation of both Cx36 gating and trafficking through PKA and Epac2, respectively [75]. From a mechanistic perspective, together with the synergistic effect on oscillations, this finding supports the view that cAMP-elevating agents could be used in conjunction with NMDAR inhibition.

Considering our previous study with MK-801 where we used 12 mM glucose as the baseline stimulatory glucose, we also briefly addressed the effects of NMDAR inhibition at different baseline glucose concentrations. To this end, we used DXO, which showed greater potency and has greater translational relevance. S7 Fig shows that application of DXO on top of the marginally stimulatory 6 mM glucose elicited oscillatory activity, which was much lower than the activity in 9 mM glucose only. Importantly, we were never able to elicit any oscillations with 10 μM DXO at a baseline glucose of 3 or 4.5 mM (not shown). Application of the NMDAR antagonist also enhanced the behavior under supraphysiological conditions (12 mM glucose) as well, but compared to 9 mM glucose + DXO, the increase in relative active time was mainly caused by an increase in oscillation duration. This parallels with our previous observations using 12 mM glucose [35] and reflects the fact that at least in NMRI mice, beyond 12 mM glucose, beta cell activity is modulated by increasing oscillation durations, even at the expense of oscillation frequency, whereas in the subphysiological to physiological regime, both the duration and frequency increase [76].

Moreover, to assess to what degree the observed effects on [Ca^2+^]_ic_ reflect the presumed effects on membrane potential, which is the more proximal step in the stimulus-secretion cascade, we performed simultaneous recordings of membrane potential and [Ca^2+^]_ic_ changes, as described previously (S8A and S8B Fig) [23]. These indicated that the observed effects on [Ca^2+^]_ic_ indeed go hand-in-hand with the effects on membrane potential, arguing against any strong membrane potential-independent direct effect on [Ca^2+^]_ic_, for instance release from intracellular stores.

Furthermore, the effects of stronger NMDAR inhibition at a higher concentration of DXO (100 μM) and in NDMAR knock-out mice (Grin1 KO) were tested, both electrophysiologically and by means of Ca^2+^ imaging (S8C–S8G Fig). The transient phases following application of DXO clearly show that stronger NMDAR inhibition progressively prolongs the duration of [Ca^2+^]_ic_ oscillations, finally resulting in continuous bursting without clear fast [Ca^2+^]_ic_ oscillations. The same behavior could be observed in Grin1 KO mice. Importantly, because the pattern of [Ca^2+^]_ic_ oscillations changes fundamentally in these two cases, network analyses based on fast [Ca^2+^]_ic_ oscillations cannot be applied in their present form.

Finally, to check whether NMDAR inhibition could have any direct effect on exocytosis, the most distal step in the stimulus-secretion coupling cascade, we performed measurements of exocytosis upon photo-release of caged Ca^2+^, both upon application of DXO and in Grin1 KO mice (S9 Fig). Findings from these experiments strongly suggest that the effects of NMDAR inhibition in beta cells are limited to the proximal membrane potential and medial [Ca^2+^]_ic_ oscillations described above.

## Discussion

Our results show that NMDAR inhibition results in increased, more regular, more synchronized, and spatiotemporally more stable beta cell activity. The increased and more regular activity is a result of both more frequent and longer fast [Ca^2+^]_ic_ oscillations, in turn yielding longer active times, together with decreased inter-oscillation interval variability. The more synchronized activity, reflected by the increased coactivity and consequently increased functional connectivity, was not a consequence of greater wave velocity which would ensure a better overlap between oscillations in different cells, or a consequence of bigger wave sizes, i.e., more cells being recruited in each wave. It was a result of more stable and coherent spatiotemporal behavior resulting in a greater similarity between subsequent waves. The latter was at least in part due to greater persistence of initiator cells in their role, as well as due to preserved paths of spreading waves.

Intracellular Ca^2+^ concentration changes can readily be recorded using Ca^2+^-sensitive fluorescent dyes in different experimental preparations, from isolated beta cells, islets of Langerhans *in vitro* and *in vivo*, in islets in an exteriorized pancreas *in vivo*, to fresh pancreatic tissue slices [4,5,77,78]. Based on these measurements, a strong and concentration-dependent causative correlation between glucose concentration, proximal electrical activity, [Ca^2+^]_ic_ changes, and distal insulin secretion has been established [23,79–81]. With increasing glucose concentrations, the membrane potential bursts and fast [Ca^2+^]_ic_ oscillations, as well as bursts of insulin secretion become more frequent and/or of longer duration [76,82,83]. After activation, the Ca^2+^ influx through NMDARs increases which is believed to subsequently activate small conductance calcium-activated potassium channels (SK4), as well as ATP-dependent potassium channels (K_ATP_) [84], thereby increasing the hyperpolarizing K^+^ conductance, hindering further plasma membrane depolarization, and promoting repolarization. While the effect of Ca^2+^ on SK4 channels is direct, the effect on K_ATP_ is probably indirect, involving NO and cGMP as second messengers and possibly other pathways [16,35,36,67,85,86]. This is expected to shorten membrane potential bursts, bring about a shorter active time, and thus inhibit insulin secretion [36]. In effect, NMDARs may constitute a local negative feedback loop preventing excessive beta cell activation by limiting the duration of bursts of depolarization [36].

In our hands, the effect of NMDAR inhibition on fast [Ca^2+^]_ic_ oscillations seemed to be very specific. We confirmed this by comparing the experimental protocol consisting of a long control stimulation with 9 mM glucose only, in order to account for the effects caused by prolonged stimulation. In the control protocol, the frequency of fast oscillations increased, but at the same time, their duration decreased. This resulted in only a moderate increase in active time by about 20%. On the other hand, when beta cells were exposed to 9mM glucose + MK-801, both the frequency and duration of fast oscillations increased, resulting in a significantly larger increase in active time of about 50% (Fig 2F). The latter observation is consistent with previous findings, where increased beta cell activity and prolongation of time spent in the depolarized state (i.e., active time) were linked with higher insulin secretion levels, even though supraphysiological glucose levels were used [35]. Taken together, this previous study and our current results obtained by employing different baseline glucose concentrations suggest that NMDAR inhibition becomes apparent in terms of [Ca^2+^]_ic_ oscillations at 6 mM glucose and higher and that the effect on oscillation duration is independent of the stimulatory concentration used, at least in the physiological (9 mM) and slightly supraphysiological (12 mM) range. Regarding prolonged stimulation with glucose only, it is worth pointing out that the effect of time spent in 9 mM glucose, at least on the time scale used in our study, differs from the effect of increasing the concentration of glucose. More specifically, we found that in the concentration range 9→12 mM glucose, higher stimulation is characterized by both longer duration and higher frequency of oscillations, whereas during prolonged stimulation with 9 mM glucose in this study, the frequency increased with time while the duration of [Ca^2+^]_ic_ oscillations decreased. This finding is practically important and worth exploring further for at least two reasons. To detect the mechanistic substrate of this intriguing phenomenon and to always account for it in studies involving long control and experimental stimulations.

Examining the collective beta cell activity revealed that the cells become more synchronized under the influence of MK-801, as reflected by a higher average coactivity coefficient and an increase in the number of functional connections in the corresponding coactivity network (Fig 2G and 2H). In contrast, beta cell synchronicity declined very convincingly in the control protocol with prolonged glucose stimulation only. Interestingly, it appears that the increase in functional connectivity under NMDAR inhibition is rather non-specific regarding the role of cells within the islets. Both well-connected cells and cells with fewer connections displayed approximately the same relative increase in node degrees (see S3 Fig). This finding is important regarding studies on heterogeneity and functional specialization within the islet beta cell [87,88]. More specifically, it has been suggested that beta cells differ at many different organizational levels, from the expression of specific genes to their functional maturity in terms of signal synchronization and insulin secretion [5,10,87,89–95]. Under NMDAR inhibition, the functional connectivity increased irrespective of a cell´s role as a very well-connected or less well-connected cell, which suggests that MK-801 exerts its effect rather homogeneously across the beta cell population, or the enhancement of intercellular signal propagation in cells more susceptible to the drug is enough to bring about a global increase in functional connectivity. It is also worth pointing out that we also analyzed whether a cell´s role in the functional network changed with prolonged stimulation with glucose or under MK-801. There was a strong positive correlation between a cell´s number of connections between intervals 1 and 2 for both the G and MK protocol (S3 Fig). Finally, we wish to point out that the increase in synchronicity observed in the present study is not necessarily and possibly not at all due to increased gap-junctional coupling. The electrotonic spread determining wave velocity and size in islets is limited by both the junctional conductance and the input resistance, the latter being set mostly by the K^+^ permeability [23,96]. Given that NMDAR inhibition most probably increases this resistance and thus increases the efficiency of junctional currents coming from neighbouring cells at depolarizing a given cell, wave velocity and the number of cells participating in a wave could be expected to increase even without a change in junctional conductance [4]. Since we did not find an increase in either wave velocity or size, this suggests that the junctional conductance did not change upon acute exposure to MK-801. However, we cannot rule out the option that a longer exposure or preincubation *in vitro* or chronic application *in vivo* would be able to affect gap-junctional coupling. We also performed additional experiments in NMDAR KO mice (see S8 Fig), but the pattern of oscillations in these mice is such that it does not allow for the same analyses as used in the present study. Possible effects of NMDAR inhibition on junctional coupling seem unlikely in our hands, but this requires further clarification in future studies, for example by means of studying changes in expression or fluorescence recovery after photobleaching [75,97]. Moreover, our current study importantly complements our previous research showing that beta cells naturally increase synchronization when exposed to progressively higher levels of glucose [98]. At a given level of glucose, this increase is probably only temporary, since synchronization seems to decrease after a longer period of glucose stimulation (Fig 2G and 2H). This finding could help explain why normal insulin secretion is impaired after prolonged periods of elevated blood glucose, as is typical in T2DM. However, we should keep in mind that the behavior in constantly elevated glucose, such as in the current, many previous studies, as well as presumably in T2DM, differs strongly from the more physiological response during oscillatory stimulation [26,99–102]. Moreover, in addition to glucose, increased serum free fatty acids, a proinflammatory milieu characterized by elevated cytokines, as well as increasing age probably contribute to the disruption of beta cell connectivity in T2DM [30,34,103–107].

Global calcium waves are the main synchronizing mechanism aligning fast calcium oscillations in beta cells, as well as in many other tissues [4,23,24,64,82,108,109]. Thus, they are the usual suspect for the observed increase in synchronicity, but our analyses of calcium wave velocities and sizes surprisingly revealed that neither was substantially affected by MK-801. We then further explored the patterns of [Ca^2+^]_ic_ wave propagation in more detail, with special focus on wave initiation and inter-wave similarity. For this purpose, we utilized multilayer network approaches and calculated the similarity between subsequent waves. It turned out that under the action of MK-801, subsequent waves followed very similar paths, as reflected by significantly higher rates of inter-wave similarity. While in glucose only, the similarity of waves was a clearly decreasing function of temporal distance in both intervals, the addition of MK-801 increased the overall similarity between waves and also lowered the decay of the inter-wave similarity curve (Fig 4). We also calculated the relative change in inter-wave similarity and it appears that MK-801 has a significant effect on the similarity between temporarily remote waves. Moreover, evaluation of the stability of wave initiators revealed that the higher degree of inter-wave similarity can at least partly be attributed to a reduced heterogeneity of initiator regions [103]. Above, we argued that cell connectivity does not seem to be affected selectively by NDMAR inhibition. Since MK-801 strongly and reproducibly affected the behavior of initiators, this might lead one to the conclusion that the drug may quite selectively target this population of cells. However, MK-801 not only stabilized the positions of initiators but the whole paths of [Ca]_ic_ waves downstream from initiators. Clearly, further mechanistic studies are required to dissect this intricate effect into more detail. However, as a possible explanation we wish to propose that by decreasing potassium permeability (and possibly other effects), MK-801 may to a relatively greater extent increase the excitability of already most excitable cells [103,110,111]. The more stable paths of waves go hand in hand with the decreased variability in inter-oscillation intervals and they could be explained by an influence of MK-801 upon stochastic channel noise [112–114]. Finally, we wish to point out that in our hands, NMDAR inhibition preserved heterogeneity between cells. Some cells still had significantly more connections than others did and in terms of wave initiation, differences between cells became even more strongly pronounced than in control conditions. Given the importance of beta cell heterogeneity and intercellular connectivity for normal islet function and glucose tolerance and their deterioration in T2DM, enhancing active time and connectivity while preserving intercellular heterogeneity seems to be an attractive pharmacological strategy for the future.

In sum, NMDAR inhibition seems to increase the time that the beta cells spend in a depolarized state by lowering the negative feedback signal which tends to repolarize beta cells once they depolarize, in turn increasing the frequency and duration of [Ca^2+^]_ic_ oscillations, in a manner which differs from cAMP-elevating agents and synergistically with them. Further, NMDAR inhibitors probably do not affect the most distal step in stimulus-secretion coupling, i.e., exocytosis. Although NMDAR inhibitors do not directly change intercellular [Ca^2+^]_ic_ wave velocity or size, they increase synchronicity between cells by virtue of lowering inter-oscillation interval variability, stabilizing wave initiators, and wave paths. It should be noted that such a precise examination of the collective function of beta cell populations could not be obtained without the novel multilayer network analysis, in which the courses of intercellular signals are encoded in weighted and directed networks. We firmly believe that assessing individual Ca^2+^ waves as network layers offers a well-founded framework for the evaluation of multicellular dynamics, even beyond the pancreatic islets. Moreover, the positive effect of NMDAR inhibition on intercellular connectivity described here importantly adds to the list of pleiotropic beta cell effects of this class of drugs [36,115]. Clearly, the results of this study shall be validated in the future on human tissue and in models of T2DM.

## Materials and methods

### Ethics statement

The study was conducted in strict accordance with all national and European recommendations pertaining to work with experimental animals and any possible animal suffering was minimized. Experimental protocol was approved by the Administration for Food Safety, Veterinary Sector and Plant Protection of the Republic of Slovenia (permit number: U34401-35/2018-2).

### Animal model and tissue slice preparation

Acute pancreatic tissue slices were prepared from 8–25 week-old male NMRI, C57BL/6J, and NMDAR KO (Grin1 KO) mice as published previously [4,116]. Briefly, after sacrificing the animals, the abdomen was accessed via laparotomy and the common bile duct was injected with low-melting point agarose 1.9% (Seaplaque GTG-agarose, Lonza Rockland Inc., Rockland, Maine, USA) in extracellular solution (ECS, consisting of (in mM) 125 NaCl, 26 NaHCO_3_, 6 glucose, 6 lactic acid, 3 myo-inositol, 2.5 KCl, 2 Na-pyruvate, 2 CaCl_2_, 1.25 NaH_2_PO_4_, 1 MgCl_2_, 0.5 ascorbic acid) at 38–40°C. Following injection, the pancreas was cooled with an ice-cold ECS and extracted. Small blocks of tissue (0.1–0.2 cm^3^ in size) were cut and transferred to a 5 ml Petri dish filled with agarose at 38–40°C and immediately cooled on ice. Cubes were sliced with the VT 1000 S vibratome (Leica, Nussloch, Germany) at 0.05 mm/s and 70 Hz into 140 mm-thick slices. Throughout preparation and slicing the tissue was kept in an ice-cold ECS continuously bubbled with a gas mixture containing 95% O_2_ and 5% CO_2_ at barometric pressure to ensure oxygenation and a pH of 7.4. The cut slices were kept in HEPES-buffered saline at room temperature (HBS, consisting of (in mM) 150 NaCl, 10 HEPES, 6 glucose, 5 KCl, 2 CaCl_2_, 1 MgCl_2_; titrated to pH = 7.4 using 1 M NaOH) until and after the dye loading. All chemicals were obtained from Sigma-Aldrich (St. Louis, Missouri, USA) unless otherwise specified.

### Dye loading

Up to 20 slices were incubated in a 5 ml Petri dish with 3.333 mL of HBS containing 6 μM calcium dye Calbryte 520 AM (AAT Bioquest, 520 Mercury Dr, Sunnyvale, California), 0.03% Pluronic F-127 (w/v), and 0.12% dimethylsulphoxide (v/v) for 50 minutes on an orbital shaker (50 turn/min) at room temperature, while being protected from light. As described earlier, the dye uptake was limited to the superficial 2–3 cell layers in the slices [4] and was not uniform among cells probably due to differences in loading-vs.-extrussion of the dye, enzyme activity, and cell viability. However, the differences in fluorescence intensity did not affect the [Ca^2+^]_ic_ time profiles therefore the relative changes in the fluorescence levels were recorded resulting in time profiles of [Ca^2+^]_ic_ in individual cells. After staining, the slices were kept in HBS (changed every 2 hours) for up to 12 hours in darkness at room temperature.

### Calcium imaging

For imaging experiments, we transferred individual slices into a bath chamber (Luigs & Neumann, Ratingen, Germany) at 37°C which was continuously superfused with bubbled (5% CO2, 95% O2) ECS. Imaging was performed on a Leica TCS SP5 AOBS Tandem II upright confocal system using a Leica HCX APO L water immersion objective (20 x, NA 1.0). Calbryte was excited with an argon 488 nm laser and the fluorescence detected by Leica HyD hybrid detector in the range of 500–700 nm (all from Leica Microsystems GmbH, Wetzlar, Germany). Images were acquired at a frequency of 10 Hz with 8-bit 256 X 256 pixels resolution at the tissue depth of around 15 μm to avoid the potentially damaged superficial cells. The thickness of the optical section was 4 μm to assure recording from a single cell layer. Before and after recording any time series, a high-resolution image (1024 X 1024 pixels) was taken for motion artefact assessment and region of interest (ROI) selection during analysis.

### Imaging of [Ca^2+^]_ic_ and whole-cell patch-clamp measurements of membrane potential oscillations

For the measurement of [Ca^2+^]_ic_ in conjunction with the patch-clamp electrophysiology, the imaging was done with a water-cooled CCD camera Andor DV 887AC-FI (Ixon, Andor Technology, Belfast, UK) mounted on an upright Nikon Eclipse E600 FN microscope (Nikon, Tokyo, Japan). OGB-1 was excited at 488 nm with a monochromator (Polychrome IV, TILL Photonics). Monochromatic light was reflected by a dichroic mirror (405 nm) and directed through a 60x water immersion objective (NA = 1.0). The emitted fluorescence was transmitted by the dichroic mirror and further filtered through a 520-nm long pass filter. Images (256 x 256 pixels) were taken at a frequency of 2 Hz (light exposure time 100 ms per image). For whole-cell patch-clamp measurements, pipettes were pulled from borosilicate glass capillaries (GC150F-15, Harvard Apparatus, USA) using a horizontal pipette puller (P-97, Sutter Instruments, USA). The pipette resistance was 2–3 MΩ in K^+^- based solution. Fast pipette capacitance (C_fast_) was compensated before, and slow membrane capacitance (C_slow_) as well as series conductance (G_s_) were compensated after whole-cell breakthrough. Only experiments with G_s_>50 nS were analyzed. Recordings were performed in the standard whole-cell mode via a patch-clamp lock-in amplifier (SWAM IIc, Celica, Slovenia) connected to a PC via A/D converter (16 bit, NI USB-6341, X Series Multifunction DAQ, National Instruments, USA) and recorded on the PC hard disk using WinFluor V3.4.1 software (John Dempster, University of Strathclyde, UK) at a sampling rate of 10 kHz. The same software was used to apply voltage protocol for identifying beta cells by their Na^+^ current inactivation properties [117] and for measuring membrane potential oscillations during stimulation with glucose and DXO. The pipette solution used for membrane potential measurements was composed of (in mM) 125 potassium methanesulfonate, 20 KCl, 40 HEPES, 2 MgCl_2_, 5 Na_2_ATP, titrated to pH = 7.2 using 1 M KOH. Osmolality was 300±10 mOsm.

### Slow photolysis experiments

Fura 6F (0.1 mM in pipette solution; Molecular Probes, USA) was used to measure [Ca^2+^]_ic_ simultaneously with the patch-clamp recordings. Fura 6F was excited at 380 nm with a monochromator (Polychrome IV; TILL Photonics, Germany). A long-pass dichroic mirror reflected the monochromatic light to the perfusion chamber and transmitted the emitted fluorescence above 400 nm, which was further filtered through a 420-nm barrier filter. The fluorescence intensity was measured with a photodiode (TILL Photonics, Germany). [Ca^2+^]_ic_ was calculated as described previously [118].

### Stimulation protocols

The control stimulation protocol consisted of sub-stimulatory 6 mM glucose in carbogen-bubbled ECS for the first 2 minutes, followed by 9 mM glucose in ECS for 40 minutes. The NDMAR antagonist test protocol consisted of sub-stimulatory 6 mM glucose for the first 2 minutes, followed by 9 mM stimulatory glucose for 20 minutes and 9 mM of stimulatory glucose + 10 μM MK-801 for the next 20 minutes [119,120].

### Processing of Ca^2+^ traces and beta cell activity analysis

The calcium time series from all manually selected ROIs were exported employing Leica Application Suite Advanced Fluorescence software (Leica Microsystems GmbH, Wetzlar, Germany). The fluorescence signals of Calbryte 520 AM were expressed as the *F*/*F*_0_ ratio. Photobleaching was accounted for by a combination of linear and exponential fitting [4]. If traces were deemed unusable (extensive motion artefacts, inactive cells etc.), they were rejected. After that, the individual signals were first band-pass filtered (between 0.05 Hz and 1.0 Hz) in order to remove baseline trends and noise, and to extract the fast oscillatory component [99]. Prior to binarization, the traces were additionally smoothed with an adjacency averaging procedure. The binarized signal was attained by setting the values from the onset to the end of individual oscillations to 1, and values between the oscillations to 0. These signals were used for the extraction of signaling parameters: oscillation frequencies and durations, relative active time, inter-oscillation interval variability, average coactivity, and the node degree in functional co-activity networks. Specifically, the average oscillation frequency of an individual cell was determined on the basis of the number of oscillations in the given time interval and the average oscillation duration of an individual cell was calculated by taking the average time of all oscillation lengths of the cell. The inter-oscillation interval variability is based on variability of the time intervals between subsequent oscillations [121].

To assess the synchronicity of the cells, we calculated the so-called coactivity parameter *CA* of the *i*-th and *j*-th cell as follows [56,122]:

$$CA_{ij}=\frac{\sum cb_{i}\left(t\right)cb_{j}\left(t\right)}{\sqrt{t_{i}^{\mathrm{tot}}t_{j}^{\mathrm{tot}}}};\;\{\begin{array}{c} cb_{i}\left(t\right)cb_{j}\left(t\right)=1;\;cb_{i}\left(t\right)\mathrm{and}\;cb_{j}\left(t\right)=1\\ cb_{i}\left(t\right)cb_{j}\left(t\right)=0;\;cb_{i}\left(t\right)\mathrm{or}\;cb_{j}\left(t\right)=0 \end{array},$$ (1)

where *cb*_*i*_(*t*) and *cb*_*j*_(*t*) are the binarized calcium time series of the *i*-th and *j*-th cell at time *t* and $t_{i}^{tot}$ and $t_{j}^{tot}$ are the total active times of the *i*-th and *j*-th cell, respectively. Using Eq (1) we constructed a coactivity matrix that contains the information about the level of synchronization between all cell pairs. To describe the global level of synchronization in the islet, we calculated the mean coactivity by averaging over all cell pairs. The coactivity matrices were then thresholded to construct functional coactivity networks, separately for all recordings and intervals. In these networks, individual cells represent nodes and connections between the *i*-th and *j*-th cell were established if *CA*_*ij*_ was higher than a pre-set threshold *CA*_th_ [25]. In addition, we calculated the temporal evolution of the average oscillation frequency, average oscillation duration and average coactivity to characterize the temporal evolution of these signaling parameters. Specifically, we used a sliding window of 180 s and a window overlap of 90 s (Fig 2).

### Multilayer network analysis of Ca^2+^ wave propagation

Calcium waves were extracted from binarized signals and each individual calcium wave represents one layer of the multiplex network. Cells *i* and *j* were deemed to be part of the same network layer *α* if the following criteria were met:

- $|T_{i}^{\alpha }-T_{j}^{\alpha }|<T_{th}$,
- *d*_*i*,*j*_<*R*_th_,
  where $T_{i}^{\alpha }$ and $T_{j}^{\alpha }$ is the oscillation onset time of the *i*-th and *j*-th cell, respectively, in the *α*-th wave, *T*_th_ a pre-set threshold time (0.5 s), *d*_*i*,*j*_ the Euclidian distance between the *i*-th and *j*-th cell and *R*_th_ a pre-set threshold distance between cells that was determined on the basis of the average intercellular distances. Directed or undirected connections were established between two cells of the same wave:
- $|T_{i}^{\alpha }-T_{j}^{\alpha }|=\Delta T_{i,j}^{\alpha }=0$ ……. undirected connection,
- $T_{th}\geq |T_{i}^{\alpha }-T_{j}^{\alpha }|=\Delta T_{i,j}^{\alpha }>0$……. directed connection.

If two cells activated in sequence (condition iv)), the direction of the connection was from the cell that activated first to the cell that activated second. The weights of the connections correspond to time delays ($\Delta T_{i,j}^{\alpha }$) in activation for a given cell pair in the *α*-th wave. With this procedure, we detected and labeled all waves within a recording. In our analyses we only used waves in which more than 45% of the islet participated. The relative size of waves was computed as the relative fraction of active cells in the given wave. For all detected waves, we computed the weighted shortest path(s) between the initiator cell(s) and the last cell(s) that were activated in the given wave. This quantity encloses the information of the physical distance traveled by the wave as well as the duration (sum of all connection weights in the shortest path), and was therefore used to compute the calcium wave velocity. However, to avoid overestimation of wave propagation velocities, because waves may originate from deeper regions of the tissue slice [24], we omitted the first two connections from the initiator. For each recording, the average velocity was computed, separately for intervals 1 and 2.

Multiplex networks were constructed for all waves in both intervals, both protocols and for all recordings. Construction was done by stacking all waves on top of one another in chronological order. This gives a visual representation of the overall appearance (similarities and differences) of the waves in the same recording. Each layer of the network contains a set of connections reflecting the path of the wave. Using these sets, we evaluated the similarity between all pairs of waves within the same recording. We calculated the similarity *R* between the waves *α* and *α*′ as follows:

$$R_{\alpha ,\alpha^{\prime }}=\frac{CON_{\mathrm{shared}}^{\alpha ,\alpha^{\prime }}}{CON_{\mathrm{unique}}^{\alpha ,\alpha^{\prime }}},$$ (2)

where $CON_{shared}^{\alpha ,\alpha \prime }$ is the number of shared (same) connections in layers *α* and *α*′, in other words the cardinality of the intersection of the two sets of connection, and $CON_{unique}^{\alpha ,\alpha \prime }$ is the number of unique connections in both waves, in other words the cardinality of the union of the two sets of connections. This gives a measure of similarity in the interval between 0 and 1, where 0 means that two waves are completely different and 1 that waves are identical. By this means we constructed similarity matrices for all waves in the given interval, separately for both protocols. We then calculated the average inter-wave similarity as a function of inter-wave distance, i.e. the average similarity *R*(*n*) for all network pairs that were *n* layers apart:

$$R\left(n\right)=\frac{1}{m}\sum_{\alpha =1}^{m}R_{\alpha \left(\alpha +n\right)},$$ (3)

where *m* is the number of all network layer pairs that are *n* layers apart. As a final measure of wave similarity, we calculated the relative change in distance between the initiator cells of individual waves in the same recording in order to examine how the NMDAR inhibition affects wave initiation in islets.

### Statistical analyses

Statistical analyses were performed using the statistics package in SigmaPlot 11 (Systat, Software Inc., Illinois, USA). We compared groups by using the t-test for normally distributed data or the Mann-Whitney rank sum test, Kruskal-Wallis one-way analysis of variance ranks, Brown-Forsythe equal variance test, Friedman repeated measures analysis of variance on ranks or Wilcoxon signed rank test for non-normally distributed data. Exact tests for individual groups are indicated in figure legends. Significance levels are expressed on figures or in figure legends.

## Supporting information

S1 Fig Average absolute cell and coactivity parameters. Absolute average values for each islet (black dots) for protocol G (left column) and protocol MK (right column) for intervals 1 (I1) and 2 (I2). a) Average oscillation frequency, b) average oscillation duration, c) average inter-oscillation interval variability and d) average coactivity. Cyan and violet dots represent the combined average values of presented parameters for protocols G and MK, respectively. Data were pooled from the following number of mice/cells/islets: 3/1373/10 (protocol G), 5/1731/12 (protocol MK).
(TIF)

S2 Fig Calcium wave parameters. Absolute average values for each islet (black dots) for protocol G (left column) and protocol MK (right column) for intervals 1 (I1) and 2 (I2). a) Average wave velocity and b) average relative wave size. Cyan and violet dots represent the combined average values of presented parameters for protocols G and MK, respectively. Data were pooled from the following number of mice/cells/islets: 3/1280/9 (protocol G), 5/1622/11 (protocol MK).
(TIF)

S3 Fig Functional network node degree. Normalized node degree in interval 1 (I1) vs. normalized node degree in interval 2 (I2) for all cells in all islets in protocol G (panel a, cyan dots, *R*^2^ = 0.26) and protocol MK (panel b, violet dots, *R*^2^ = 0.59). Grey lines represent the linear fit. All values are normalized according to the number of cells in each individual recording. Cells without functional connections were excluded. Data were pooled from the following number of mice/cells/islets: 3/1373/10 (protocol G), 5/1731/12 (protocol MK).
(TIF)

S4 Fig Visualizing beta cell activity after stimulation with dextrorphan (protocol DXO) and forskolin (protocol FOR). Mean field signal of a representative recording with protocol DXO (9 mM glucose + 10 μM dextrorphan) (A) and protocol FOR (9 mM glucose + 10 μM Forskolin) (B). Representative [Ca^2+^]_ic_ signals and raster plots in a recording with protocol DXO (B, C) and protocol FOR (E, F). Panels B, E and C, F show interval 1 and interval 2 used for analysis, respectively. Inserts show 35 s outtakes from the time series and binarized plots.
(TIF)

S5 Fig Comparing beta cell activity in protocols G, MK, DXO and FOR. The relative change in different cellular signaling parameters from interval 1 to interval 2 for protocols G (cyan, glucose only) and MK (violet, 9 mM glucose + 10 μM MK-801) (shown previously in Fig 2 in the main manuscript), and protocols DXO (purple, 9 mM glucose + 10 μM dextrorphan) and FOR (navy, 9 mM glucose + 10 μM Forskolin): Relative change in oscillation frequency (A), relative change in oscillation duration (B), relative change in relative active time (C), relative change in inter-oscillation-interval variability (D), relative change in coactivity (E), and the relative change in node degree in the functional network (F). Whiskers indicate the 10th and 90th percentile, the box indicates the 25th and 75th percentile, and the horizontal line indicates the median value. Data were pooled from the following number of cells/islets: 1373/10 (protocol G; same data set as in the main article Fig 2), 1731/12 (protocol MK; same data set as in the main article Fig 2), 767/6 (protocol DXO), 872/5 (protocol FOR). *p*–significance level. *Differences between all pairs of data sets are statistically significant (*p*<0.001) unless otherwise indicated. ns–Differences between all pairs of data sets are statistically not significant (*p*>0.05) unless otherwise indicated. Statistical tests: Kruskal-Wallis One Way Analysis of Variance on Ranks (A, B, C, D, F) and Brown-Forsythe Equal Variance Test (E).
(TIF)

S6 Fig Beta cell activity with subsequent stimulation with glucose, dextrorphan and forskolin (protocol DXO+FOR). Average signal of a representative recording with indicated stimulation protocol (A). The three intervals of the protocol are indicated with light grey, grey and dark grey bars, respectively. The sub-stimulatory period in 6 mM glucose is indicated with dashed bars. Interval 1 (light grey, I1), interval 2 (grey, I2) and interval 3 (dark grey, I3) are shown in panels B, C and D, respectively. Panels (E)-(H) show the absolute values of different signaling parameters: oscillation frequency (E), oscillation duration (F), relative active time (G) and inter-oscillation-interval variability (H), separately for interval 1 (light grey), interval 2 (grey) and interval 3 (dark grey). Whiskers indicate the 10th and 90th percentile, the box indicates the 25th and 75th percentile, and the horizontal line indicates the median value. Data were pooled from 2 islets (230 cells) subjected to the same protocol. *p*–significance level. *Differences between all pairs of data sets are statistically significant (*p*<0.001) unless otherwise indicated. Statistical test: Friedman Repeated Measured Analysis of Variance on Ranks (E, F, G, H).
(TIF)

S7 Fig Characterization of a glucose-dependent effect of NMDAR inhibition. Representative [Ca^2+^]_ic_ signals and raster plots (A,B) in a recording with 6 mM glucose (A, left) and subsequent 6 mM glucose + 10 μM DXO (A, right) and in a recording with 12 mM glucose (B, left) and subsequent 12 mM glucose + 10 μM DXO (B, right) (B). Box-plots in panels C, D and E show absolute values of basic cellular signaling parameters for interval 1 (I1) with 6 mM (light grey), 9 mM (grey) and 12 mM (dark grey) glucose stimulation and interval 2 (I2) with 6 mM (light grey), 9 mM (grey) and 12 mM (dark grey) glucose with the addition of 10 μM DXO. Whiskers indicate the 10th and 90th percentile, the box indicates the 25th and 75th percentile, and the horizontal line indicates the median value. Data were pooled from the following number of cells/islets: 500/4 (6 mM), 767/6 (9 mM), 272/2 (12 mM). *p*–significance level. Statistical test: Wilcoxon Signed Rank Test (C, D, E).
(TIF)

S8 Fig Pharmacological inhibition using DXO and genetic ablation of NMDAR prolong the depolarization phase and plateau fraction of membrane potential and Ca^2+^ oscillations in glucose-stimulated beta cells. A) Simultaneous recording of membrane potential oscillations in a patch-clamped cell (indicated by the yellow plus sign) and [Ca^2+^]_ic_ oscillations in other beta cells of the same islet of Langerhans (indicated by colored squares). [Ca^2+^]_ic_ oscillations were measured with a CCD camera at a temporal resolution of 2 Hz in cells in the tissue slice loaded with OGB-1. Membrane potential was recorded at a temporal resolution of 10000 Hz. B) Membrane potential (black trace) and [Ca^2+^]_ic_ responses (colored traces) of cells shown in A to stimulation with 12 mM glucose followed by the addition of 10 μM DXO are presented. 2 insets represent a short interval of recording in 12 mM glucose and 12 mM glucose with 10 μM DXO, respectively. Note the tight relationship between membrane potential and [Ca^2+^]_ic_ changes. C) Representative membrane potential measurement of a single pancreatic beta cell from WT mouse exposed to 12 mM glucose and the subsequent addition of 100 μm DXO. 2 insets represent a short interval of recording in 12 mM glucose and 12 mM glucose with 100 μM DXO, respectively. Note the increase in burst duration and the progress to the so-called continuous bursting in 100 μM DXO. The cells do not turn off immediately after a switch to 6mM glucose but do so in 3 mM glucose. D) Representative membrane potential measurement of a single pancreatic beta cell from a Grin1 KO mouse exposed to 12 mM glucose. Inset represents a short interval of recording in 12 mM glucose. Note that the electrical activity is not organized in individual bursts but consists of continuous bursting, similar to 100 μM DXO. E) Representative trace of calcium recording using confocal microscope (1 Hz) after stimulation of beta cells from WT islet with 12 mM glucose and the subsequent addition of 10 μm DXO. 2 insets represent a short interval of recording in 12 mM glucose and 12 mM glucose with 10 μM DXO, respectively. F) Representative trace of calcium recording using confocal microscope (1 Hz) after stimulation of beta cells from Grin1 WT islet with 12 mM glucose and the subsequent addition of 10 μm DXO and 100 μM DXO. Inset represents a short interval of recording in 12 mM glucose after switching from 10 to 100 μM of DXO. G) Representative trace of calcium recording using confocal microscope (1 Hz) after stimulation of beta cells from Grin1 KO islet with 12 mM glucose. Note that qualitatively, islets from Bl6J mice (the genetic background of Grin1 KO mice) show the same responses to DXO as NMRI mice (used in our study) and that in high DXO and in KO mice there are no discernible fast [Ca^2+^]_ic_ oscillations due to the continuous depolarization with spiking activity (compare F and G with C and D).
(TIF)

S9 Fig Pharmacological inhibition using DXO and genetic ablation of NMDAR do not change the sensitivity of secretory machinery to calcium. A) Slow photo-release of caged Ca^2+^ produces a ramp-like increase in [Ca^2+^]_ic_ (upper panel). After reaching the threshold value of [Ca^2+^]_ic_ (Ca_C_) an increase in membrane capacitance (Cm) is triggered (lower panel). B) The rate of the C_m_ change shows saturation kinetics when plotted versus [Ca^2+^]_ic_, with high cooperativity and half-effective [Ca^2+^]_ic_ (EC_50_) at 3 mM. A Hill function was fitted through the data (*red line*). C) Box plot represents the [Ca^2+^]_ic_ needed for triggering a Cm change (Ca_C_). Median Ca_C_ values (Grin1 WT: 1^st^ quartile = 1.76 μM, median = 1.89 μM, 3^rd^ quartile = 2.18 μM, n = 8; Grin1 WT + 10 μM DXO: 1^st^ quartile = 1.62 μM, median = 2.14 μM, 3^rd^ quartile = 2.53 μM, n = 9; Grin1 KO: 1^st^ quartile = 1.83 μM, median = 1.97 μM, 3^rd^ quartile = 2.10 μM, n = 8) did not differ significantly among groups (Kruskal-Wallis test). Red dots represent mean values. D) Box plot represents the half-effective [Ca^2+^]_ic_ (EC_50_). Median EC_50_ values (Grin1 WT: 1^st^ quartile = 2.485 μM, median = 2.71 μM, 3^rd^ quartile = 2.96 μM, n = 8; Grin1 WT + 10 μM DXO: 1^st^ quartile = 2.24 μM, median = 2.94 μM, 3^rd^ quartile = 3.51 μM, n = 9; Grin1 KO: 1^st^ quartile = 2.72 μM, median = 2.85 μM, 3^rd^ quartile = 3.02 μM, n = 8) did not differ significantly among groups (One-way ANOVA and Tukey’s multiple comparisons test). Red dots represent the mean values.
(TIF)
