# Supplementary figures and images for: NMDA receptor inhibition increases, synchronizes, and stabilizes the collective pancreatic beta cell activity: Insights through multilayer network analysis
Source: PLoS Comput Biol. 2021 May 11;17(5):e1009002. doi: 10.1371/journal.pcbi.1009002 (PMC8139480; doi:10.1371/journal.pcbi.1009002)

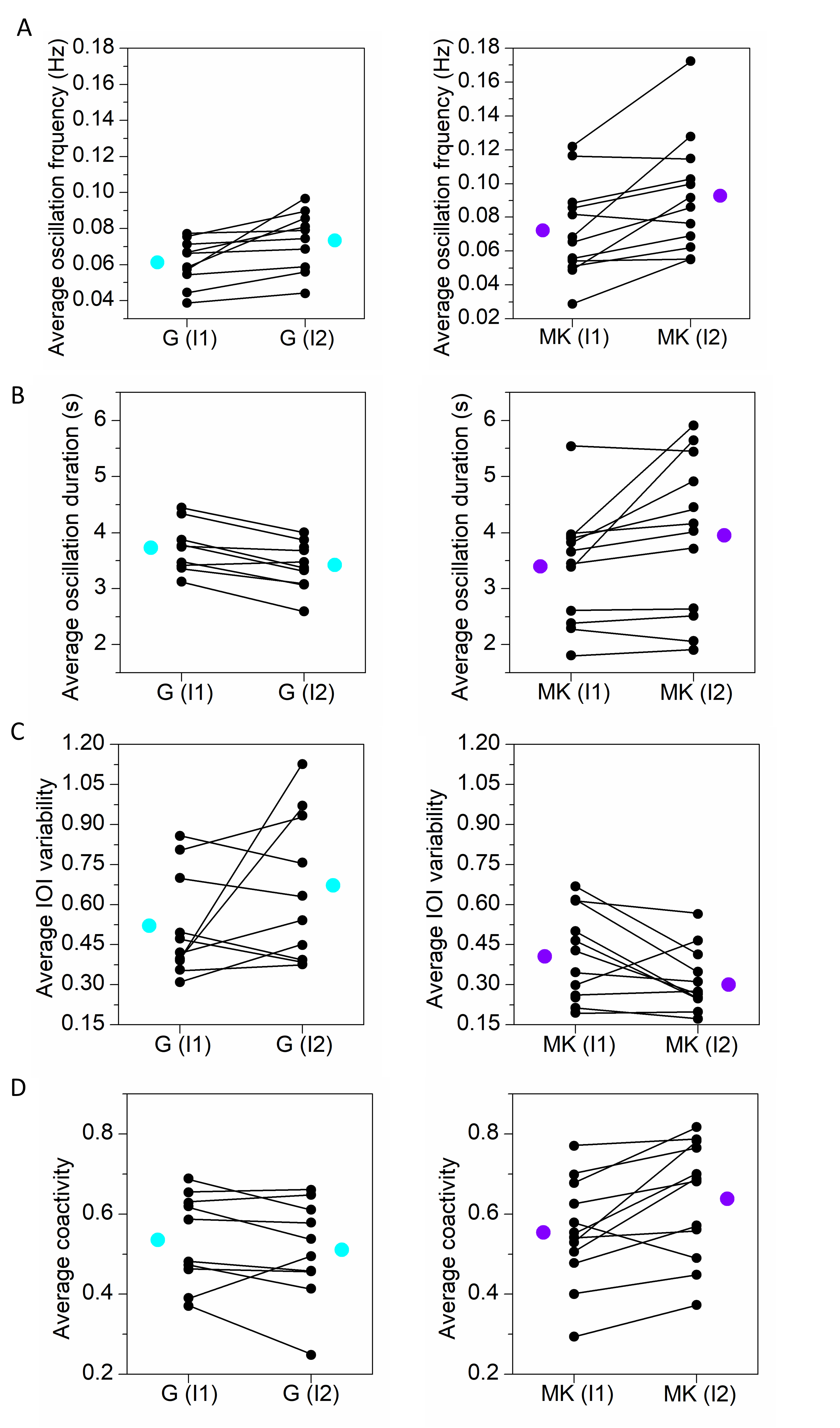

Supplement: S1 Fig — Absolute average values for each islet (black dots) for protocol G (left column) and protocol MK (right column) for intervals 1 (I1) and 2 (I2). a) Average oscillation frequency, b) average oscillation duration, c) average inter-oscillation interval variability and d) average coactivity. Cyan and violet dots represent the combined average values of presented parameters for protocols G and MK, respectively. Data were pooled from the following number of mice/cells/islets: 3/1373/10 (protocol G), 5/1731/12 (protocol MK). (TIF) [file pcbi.1009002.s001.tif]

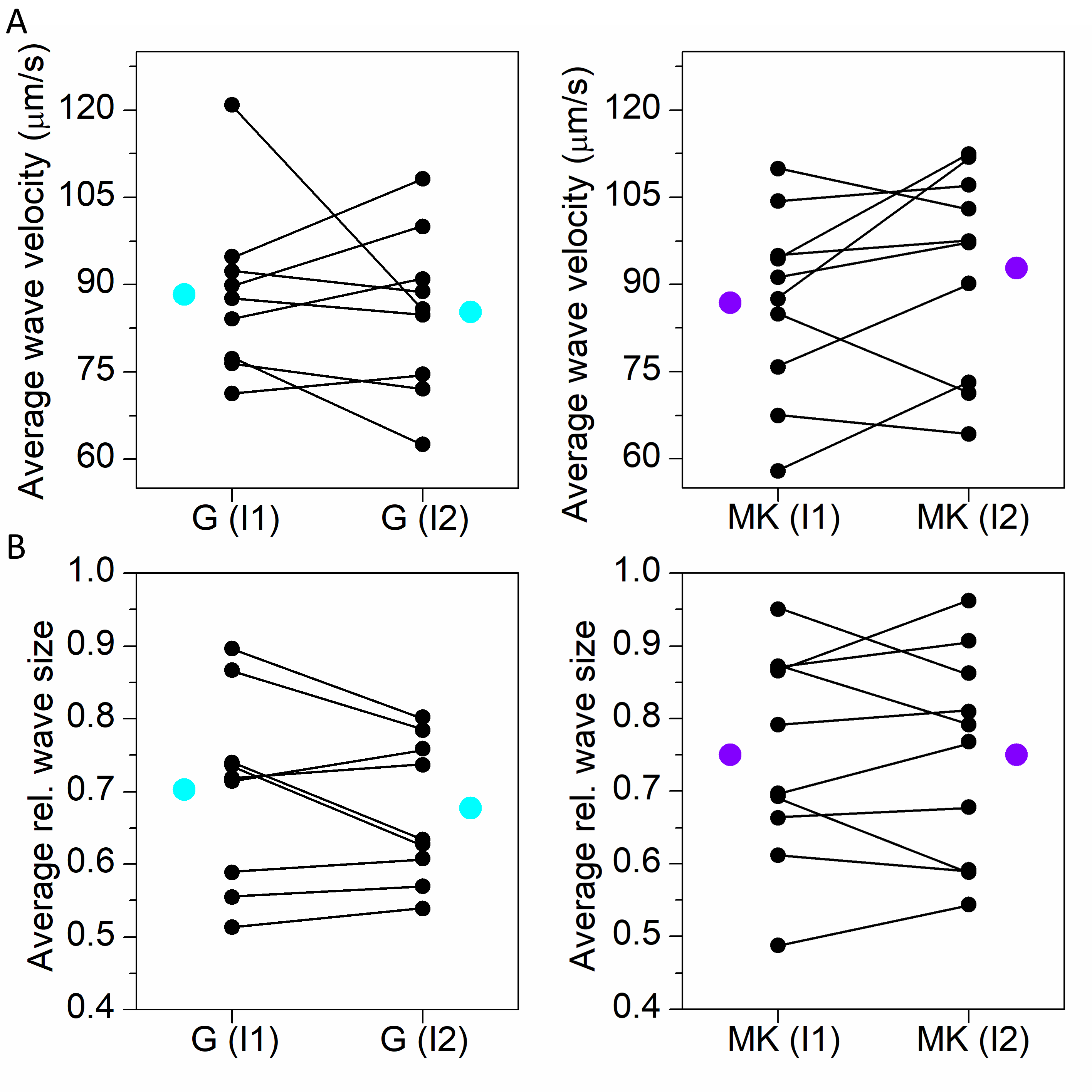

Supplement: S2 Fig — Absolute average values for each islet (black dots) for protocol G (left column) and protocol MK (right column) for intervals 1 (I1) and 2 (I2). a) Average wave velocity and b) average relative wave size. Cyan and violet dots represent the combined average values of presented parameters for protocols G and MK, respectively. Data were pooled from the following number of mice/cells/islets: 3/1280/9 (protocol G), 5/1622/11 (protocol MK). (TIF) [file pcbi.1009002.s002.tif]

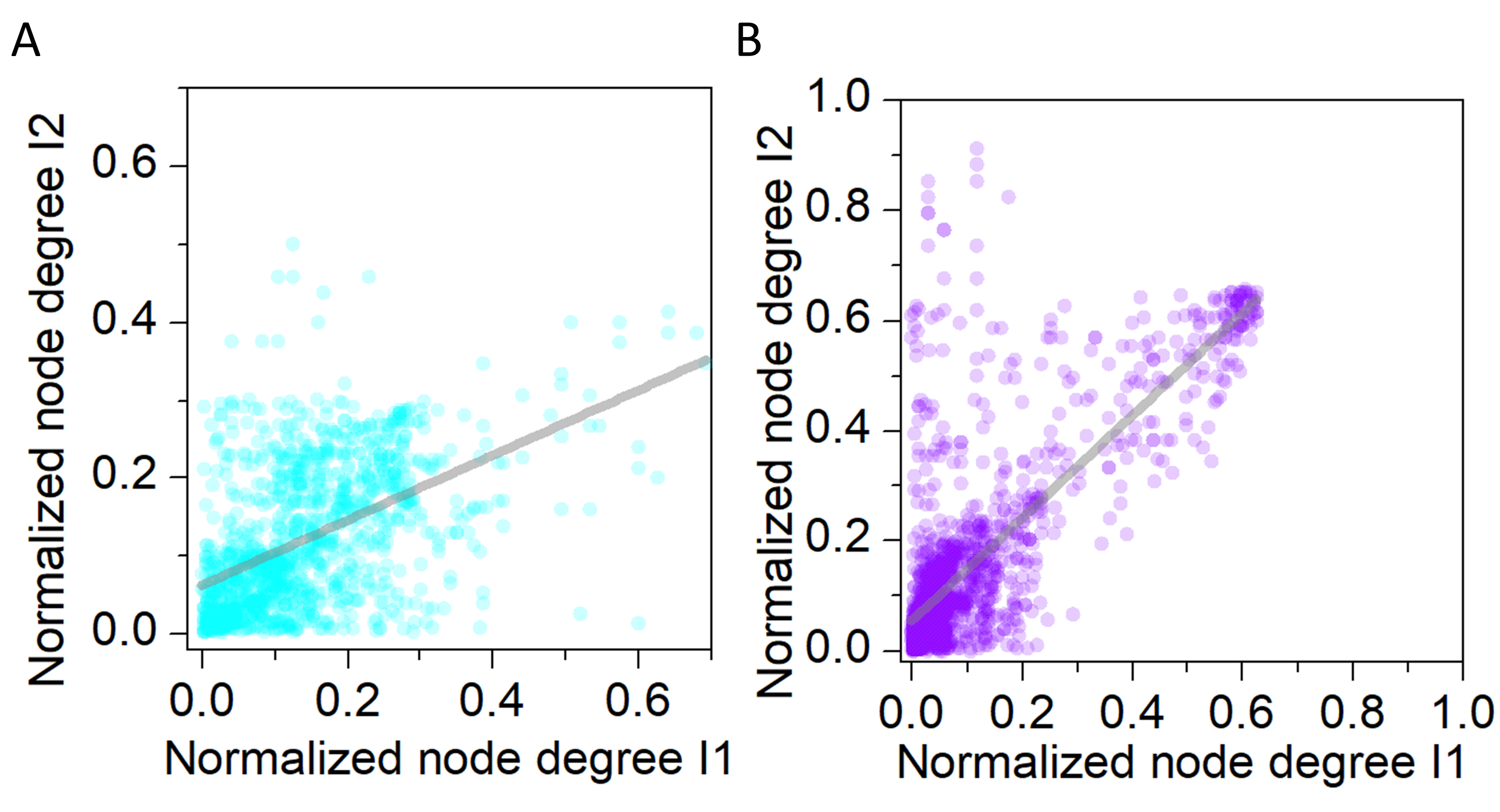

Supplement: S3 Fig — Normalized node degree in interval 1 (I1) vs. normalized node degree in interval 2 (I2) for all cells in all islets in protocol G (panel a, cyan dots, R2 = 0.26) and protocol MK (panel b, violet dots, R2 = 0.59). Grey lines represent the linear fit. All values are normalized according to the number of cells in each individual recording. Cells without functional connections were excluded. Data were pooled from the following number of mice/cells/islets: 3/1373/10 (protocol G), 5/1731/12 (protocol MK). (TIF) [file pcbi.1009002.s003.tif]

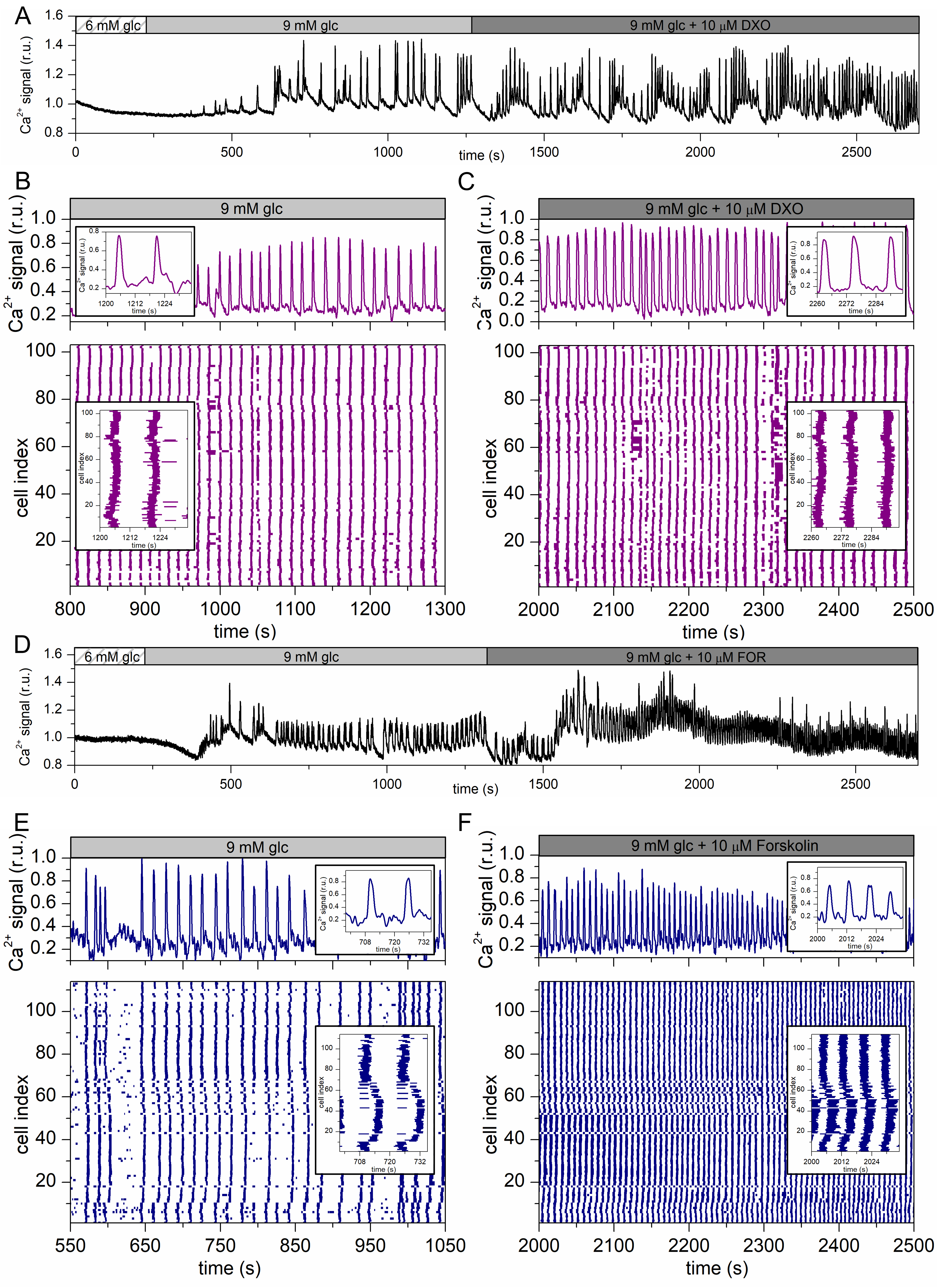

Supplement: S4 Fig — Mean field signal of a representative recording with protocol DXO (9 mM glucose + 10 μM dextrorphan) (A) and protocol FOR (9 mM glucose + 10 μM Forskolin) (B). Representative [Ca2+]ic signals and raster plots in a recording with protocol DXO (B, C) and protocol FOR (E, F). Panels B, E and C, F show interval 1 and interval 2 used for analysis, respectively. Inserts show 35 s outtakes from the time series and binarized plots. (TIF) [file pcbi.1009002.s004.tif]

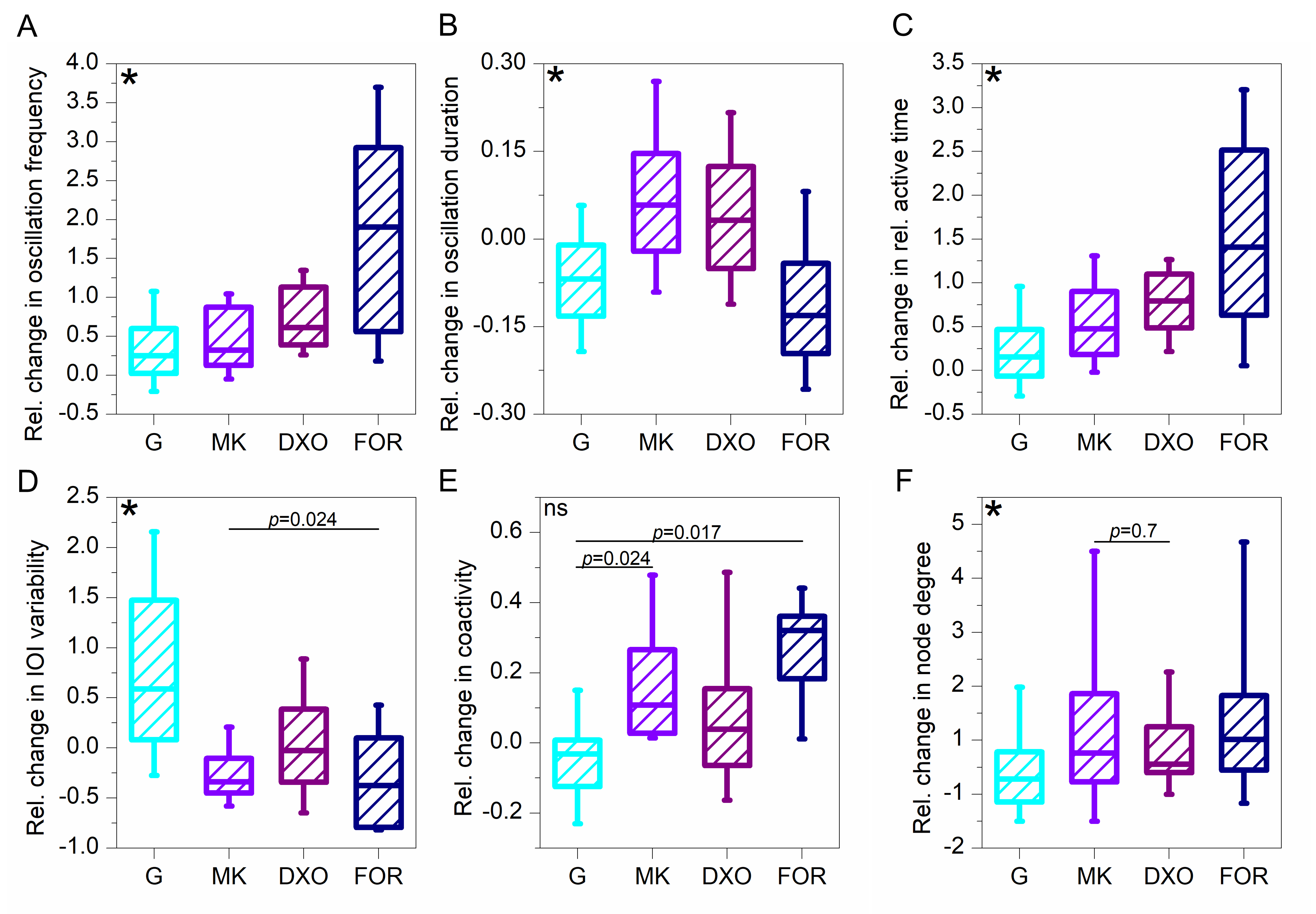

Supplement: S5 Fig — The relative change in different cellular signaling parameters from interval 1 to interval 2 for protocols G (cyan, glucose only) and MK (violet, 9 mM glucose + 10 μM MK-801) (shown previously in Fig 2 in the main manuscript), and protocols DXO (purple, 9 mM glucose + 10 μM dextrorphan) and FOR (navy, 9 mM glucose + 10 μM Forskolin): Relative change in oscillation frequency (A), relative change in oscillation duration (B), relative change in relative active time (C), relative change in inter-oscillation-interval variability (D), relative change in coactivity (E), and the relative change in node degree in the functional network (F). Whiskers indicate the 10th and 90th percentile, the box indicates the 25th and 75th percentile, and the horizontal line indicates the median value. Data were pooled from the following number of cells/islets: 1373/10 (protocol G; same data set as in the main article Fig 2), 1731/12 (protocol MK; same data set as in the main article Fig 2), 767/6 (protocol DXO), 872/5 (protocol FOR). p–significance level. *Differences between all pairs of data sets are statistically significant (p<0.001) unless otherwise indicated. ns–Differences between all pairs of data sets are statistically not significant (p>0.05) unless otherwise indicated. Statistical tests: Kruskal-Wallis One Way Analysis of Variance on Ranks (A, B, C, D, F) and Brown-Forsythe Equal Variance Test (E). (TIF) [file pcbi.1009002.s005.tif]

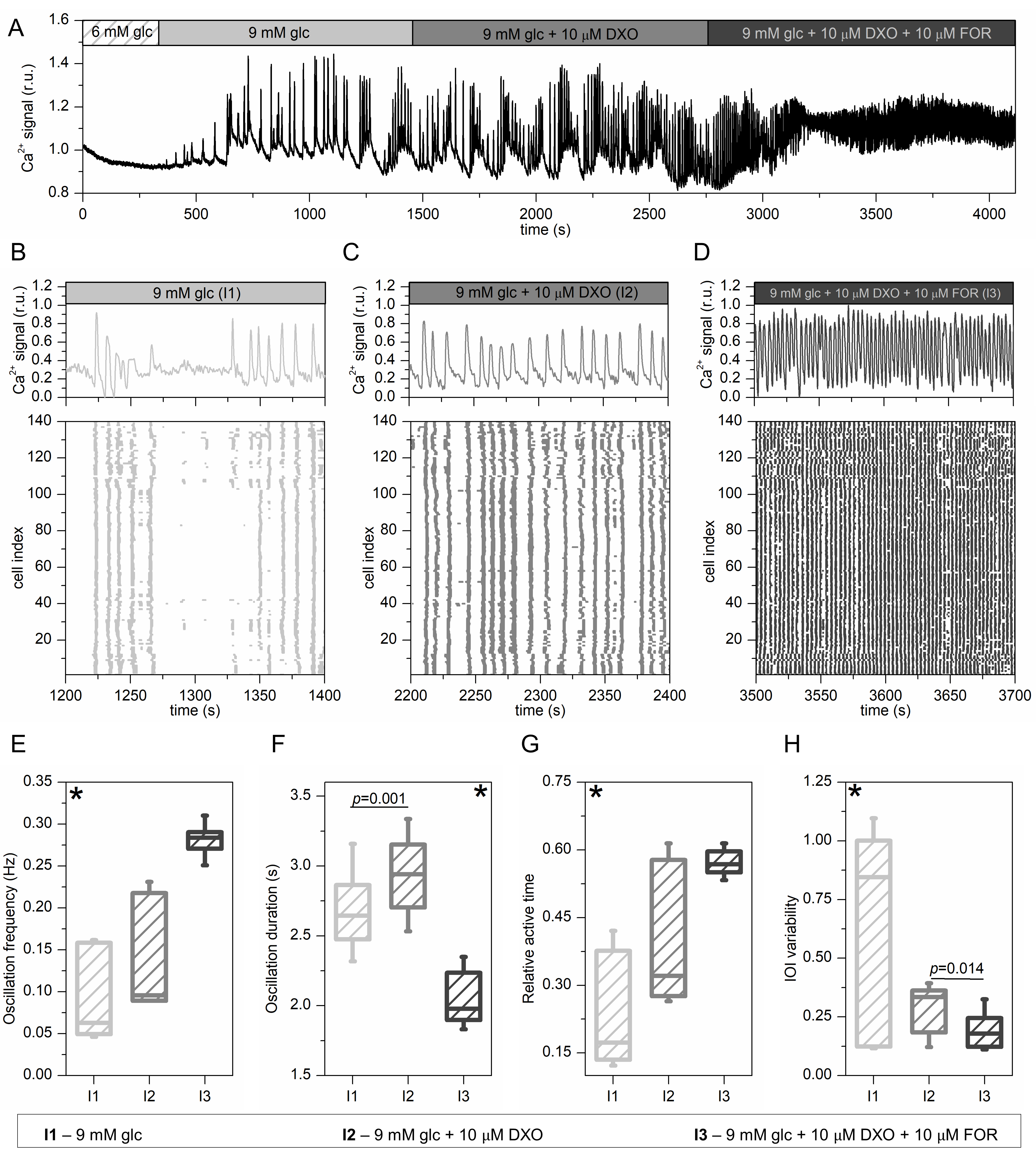

Supplement: S6 Fig — Average signal of a representative recording with indicated stimulation protocol (A). The three intervals of the protocol are indicated with light grey, grey and dark grey bars, respectively. The sub-stimulatory period in 6 mM glucose is indicated with dashed bars. Interval 1 (light grey, I1), interval 2 (grey, I2) and interval 3 (dark grey, I3) are shown in panels B, C and D, respectively. Panels (E)-(H) show the absolute values of different signaling parameters: oscillation frequency (E), oscillation duration (F), relative active time (G) and inter-oscillation-interval variability (H), separately for interval 1 (light grey), interval 2 (grey) and interval 3 (dark grey). Whiskers indicate the 10th and 90th percentile, the box indicates the 25th and 75th percentile, and the horizontal line indicates the median value. Data were pooled from 2 islets (230 cells) subjected to the same protocol. p–significance level. *Differences between all pairs of data sets are statistically significant (p<0.001) unless otherwise indicated. Statistical test: Friedman Repeated Measured Analysis of Variance on Ranks (E, F, G, H). (TIF) [file pcbi.1009002.s006.tif]

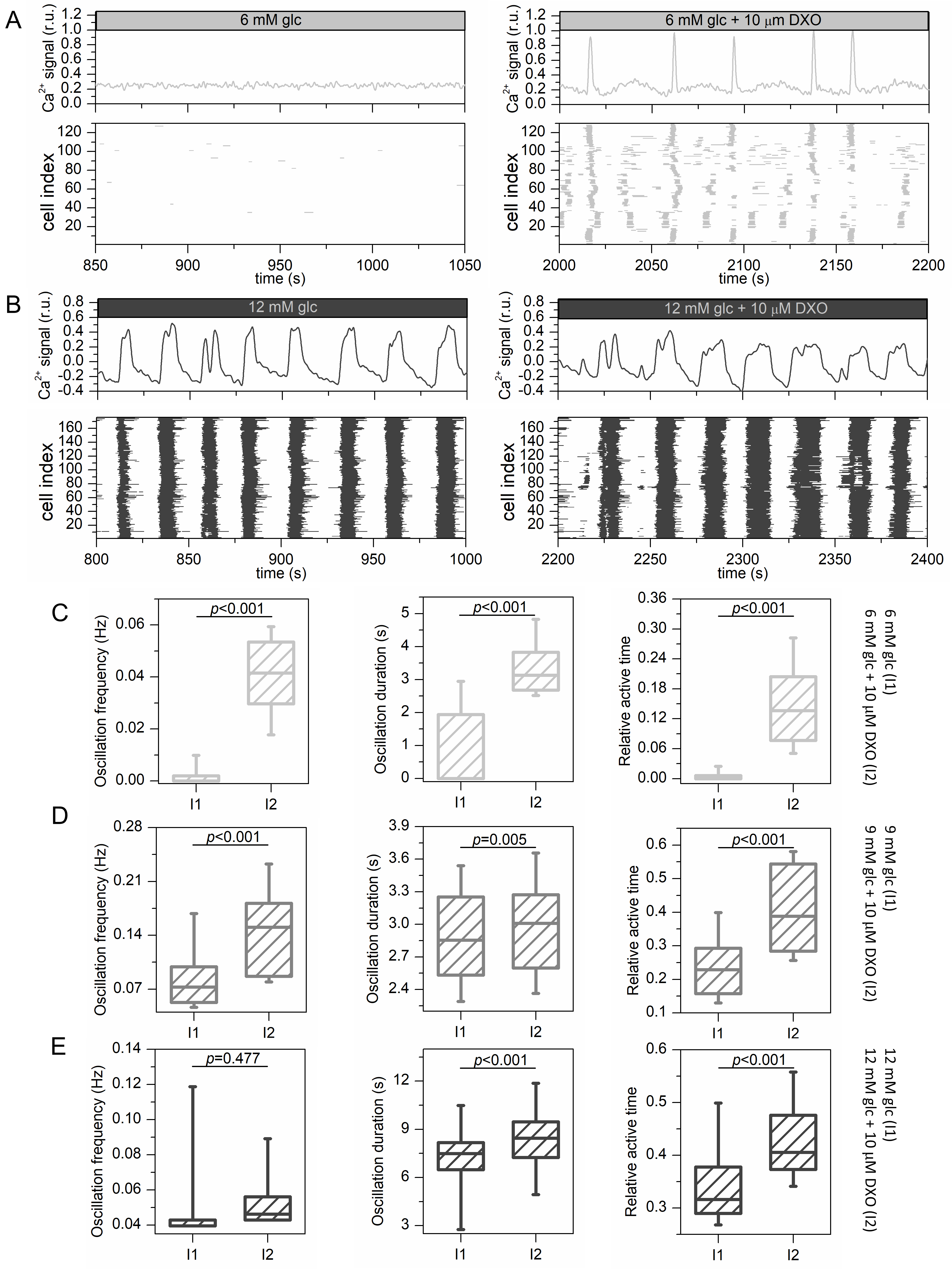

Supplement: S7 Fig — Representative [Ca2+]ic signals and raster plots (A,B) in a recording with 6 mM glucose (A, left) and subsequent 6 mM glucose + 10 μM DXO (A, right) and in a recording with 12 mM glucose (B, left) and subsequent 12 mM glucose + 10 μM DXO (B, right) (B). Box-plots in panels C, D and E show absolute values of basic cellular signaling parameters for interval 1 (I1) with 6 mM (light grey), 9 mM (grey) and 12 mM (dark grey) glucose stimulation and interval 2 (I2) with 6 mM (light grey), 9 mM (grey) and 12 mM (dark grey) glucose with the addition of 10 μM DXO. Whiskers indicate the 10th and 90th percentile, the box indicates the 25th and 75th percentile, and the horizontal line indicates the median value. Data were pooled from the following number of cells/islets: 500/4 (6 mM), 767/6 (9 mM), 272/2 (12 mM). p–significance level. Statistical test: Wilcoxon Signed Rank Test (C, D, E). (TIF) [file pcbi.1009002.s007.tif]

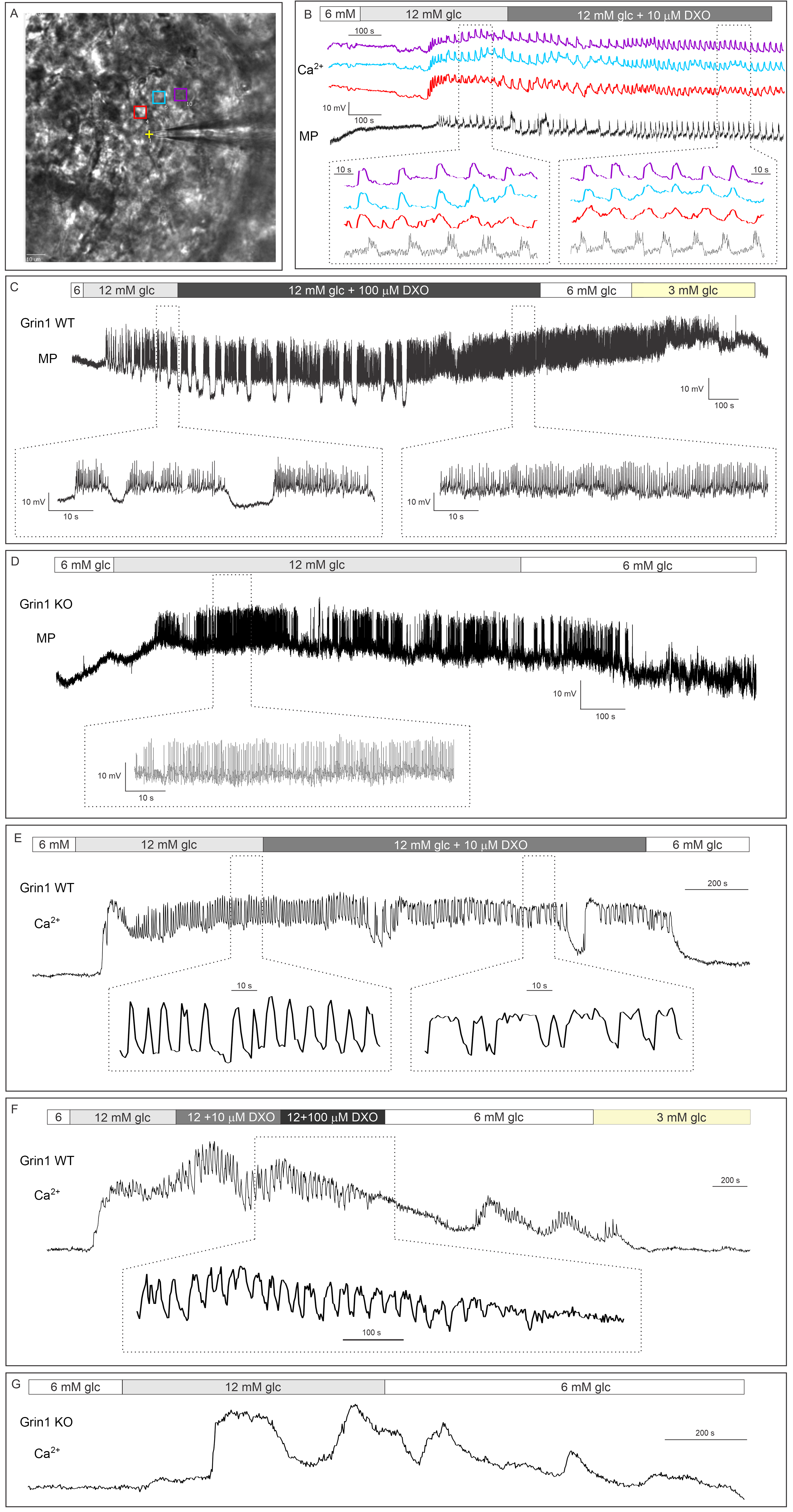

Supplement: S8 Fig — A) Simultaneous recording of membrane potential oscillations in a patch-clamped cell (indicated by the yellow plus sign) and [Ca2+]ic oscillations in other beta cells of the same islet of Langerhans (indicated by colored squares). [Ca2+]ic oscillations were measured with a CCD camera at a temporal resolution of 2 Hz in cells in the tissue slice loaded with OGB-1. Membrane potential was recorded at a temporal resolution of 10000 Hz. B) Membrane potential (black trace) and [Ca2+]ic responses (colored traces) of cells shown in A to stimulation with 12 mM glucose followed by the addition of 10 μM DXO are presented. 2 insets represent a short interval of recording in 12 mM glucose and 12 mM glucose with 10 μM DXO, respectively. Note the tight relationship between membrane potential and [Ca2+]ic changes. C) Representative membrane potential measurement of a single pancreatic beta cell from WT mouse exposed to 12 mM glucose and the subsequent addition of 100 μm DXO. 2 insets represent a short interval of recording in 12 mM glucose and 12 mM glucose with 100 μM DXO, respectively. Note the increase in burst duration and the progress to the so-called continuous bursting in 100 μM DXO. The cells do not turn off immediately after a switch to 6mM glucose but do so in 3 mM glucose. D) Representative membrane potential measurement of a single pancreatic beta cell from a Grin1 KO mouse exposed to 12 mM glucose. Inset represents a short interval of recording in 12 mM glucose. Note that the electrical activity is not organized in individual bursts but consists of continuous bursting, similar to 100 μM DXO. E) Representative trace of calcium recording using confocal microscope (1 Hz) after stimulation of beta cells from WT islet with 12 mM glucose and the subsequent addition of 10 μm DXO. 2 insets represent a short interval of recording in 12 mM glucose and 12 mM glucose with 10 μM DXO, respectively. F) Representative trace of calcium recording using confocal microscope (1 Hz) after s [file pcbi.1009002.s008.tif]

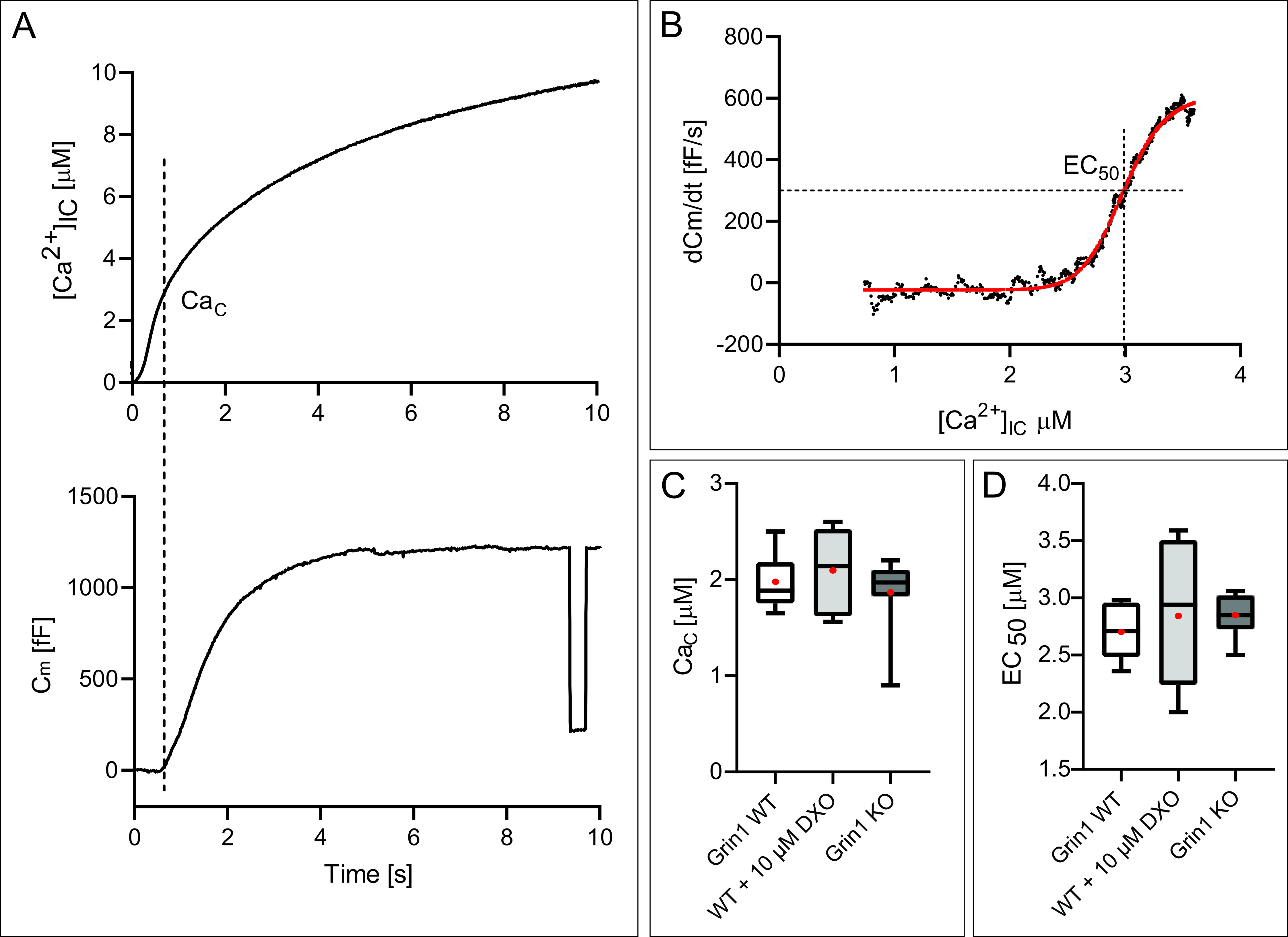

Supplement: S9 Fig — A) Slow photo-release of caged Ca2+ produces a ramp-like increase in [Ca2+]ic (upper panel). After reaching the threshold value of [Ca2+]ic (CaC) an increase in membrane capacitance (Cm) is triggered (lower panel). B) The rate of the Cm change shows saturation kinetics when plotted versus [Ca2+]ic, with high cooperativity and half-effective [Ca2+]ic (EC50) at 3 mM. A Hill function was fitted through the data (red line). C) Box plot represents the [Ca2+]ic needed for triggering a Cm change (CaC). Median CaC values (Grin1 WT: 1st quartile = 1.76 μM, median = 1.89 μM, 3rd quartile = 2.18 μM, n = 8; Grin1 WT + 10 μM DXO: 1st quartile = 1.62 μM, median = 2.14 μM, 3rd quartile = 2.53 μM, n = 9; Grin1 KO: 1st quartile = 1.83 μM, median = 1.97 μM, 3rd quartile = 2.10 μM, n = 8) did not differ significantly among groups (Kruskal-Wallis test). Red dots represent mean values. D) Box plot represents the half-effective [Ca2+]ic (EC50). Median EC50 values (Grin1 WT: 1st quartile = 2.485 μM, median = 2.71 μM, 3rd quartile = 2.96 μM, n = 8; Grin1 WT + 10 μM DXO: 1st quartile = 2.24 μM, median = 2.94 μM, 3rd quartile = 3.51 μM, n = 9; Grin1 KO: 1st quartile = 2.72 μM, median = 2.85 μM, 3rd quartile = 3.02 μM, n = 8) did not differ significantly among groups (One-way ANOVA and Tukey’s multiple comparisons test). Red dots represent the mean values. (TIF) [file pcbi.1009002.s009.tif]
